# MERS-CoV and SARS-CoV-1 proteins inhibit the IFN-α JAK/STAT pathway of epithelial cells, via IFN-λ-induced USP18

**DOI:** 10.3389/fimmu.2026.1739662

**Published:** 2026-02-19

**Authors:** Yamei Zhang, Nigel J. Stevenson

**Affiliations:** Viral Immunology Group, Trinity Biomedical Sciences Institute, School of Biochemistry and Immunology, Trinity College Dublin, Dublin, Ireland

**Keywords:** IFN-λ, interferon, MERS-CoV, SARS-CoV-1, STAT, USP18

## Abstract

The recent emergence of Severe Acute Respiratory Syndrome Coronavirus (SARS-CoV)-2 highlights the need for greater understanding of the immune evasion mechanisms used by Coronavirus (CoVs) to subvert antiviral responses. Previous global outbreaks caused by Middle East respiratory syndrome coronavirus (MERS-CoV) and SARS-CoV-1 were associated with high mortality rates and limited therapeutic options. Interferon (IFN)-α is the body’s natural antiviral agent; but its Janus kinase/signal transducer and activators of transcription (JAK/STAT) signalling pathway is often antagonized by viruses, thereby preventing the upregulation of essential, anti-viral IFN Stimulated Genes (ISGs). Notably, therapeutic IFN-α has disappointingly weak clinical responses in MERS-CoV and SARS-CoV-1 infected patients, indicating that these CoVs inhibit the IFN-α JAK/STAT pathway. We previously identified that MERS-CoV-non-structural protein(nsp)2 and nsp5 and SARS-CoV-1-nsp14 block the IFN-α JAK/STAT signalling pathway in human epithelial A549 cells; however, the mechanisms behind this inhibition remain unknown. In this study, we explored the factors influencing basal STAT1 and STAT2 phosphorylation and discovered that the expression of MERS-CoV-nsp2 and SARS-CoV-1-nsp14, but not MERS-CoV-nsp5, upregulated IFN-λ1/3 in A549 cells. Neutralization of IFN-λ1/3 revealed that this induction was responsible for the observed basal STAT1 and STAT2 phosphorylation, resulting in reduced responsiveness to exogenous IFN-α. Furthermore, both MERS-CoV-nsp2 and SARS-CoV-1-nsp14 induced the expression of USP18, a negative regulator of the IFN-α JAK/STAT pathway, resulting in reduced responsiveness to exogenous IFN-α. Silencing USP18 reinstated IFN-α-mediated STAT1 phosphorylation and ISG induction. Collectively, these findings shed light on the diverse strategies employed by these CoVs to evade type I IFN antiviral responses. While providing evidence for the ineffectiveness of exogenous IFN-α treatment during CoV infection, our discoveries also identify these viral proteins as potential targets for therapeutic intervention.

## 1 Introduction

The effective antiviral role of Interferon (IFN)-α, has been harnessed for the treatment of several viral infections, including Hepatitis B virus (HBV) (1), Hepatitis C virus (HCV) (2), Human papillomavirus (HPV) (3) and Human Herpes virus (HHV) infections (4). Unfortunately, therapeutic trials have shown that the application of IFN-α in MERS-CoV infections had disappointingly weak clinical responses, with a retrospective study finding that IFN-α2a treatment of MERS-CoV patients did not improve the recovery rate (5). IFN-α has been shown to not effectively inhibit SARS-CoV-1 replication *in vitro* and presented suboptimal responses in SARS-CoV-1 patients (6, 7). This lack of response to exogenous IFN-α suggests that these Coronavirus (CoVs) encode antagonists that counteract JAK/STAT signalling. Indeed, the nsp1 of SARS-CoV-1 has been shown to decrease STAT1 activation, and SARS-CoV-1 ORF6 protein sequesters STAT1 nuclear import factors (karyopherin alpha 2 and karyopherin beta 1) on the endoplasmic reticulum (ER)/Golgi membrane (8, 9). The SARS-CoV-1 ORF3a protein causes ER stress and induces serine phosphorylation-dependent degradation of the IFN-α receptor subunit 1 (IFNAR1) (10); while MERS-CoV ORF4a, 4b and M proteins all inhibit IFN signalling through efficiently blocking ISRE promoter activity (11).

The initiation of the JAK/STAT pathway by IFN is critical within the innate antiviral response, as it leads to the transcription of numerous ISGs, that effectively limit viral replication (12). Having previously shown that MERS-CoV-nsp2 and SARS-CoV-1-nsp14 expression in A549 epithelial cells inhibited IFN-α-mediated STAT phosphorylation, downstream ISG induction and STAT nuclear translocation (13), we postulated that these viral proteins mechanistically modulate negative regulators of the JAK/STAT pathway. The JAK/STAT pathway is regulated in several ways. At the ligand level, IFN receptors undergo downregulation or internalization to reduce signalling (14, 15). Downstream of this, Suppressor of Cytokine Signalling (SOCS) family proteins block signalling cascades, by interacting with activated JAK kinases and the receptor chains (16, 17). The Ubiquitin specific peptidase 18 (USP18), is a key negative regulator that interacts with JAK1 and inhibits downstream signals (18). Phosphatases and protein inhibitor of activated STAT (PIAS), are another two families of JAK/STAT inhibitors. Phosphatases can attenuate signalling by dephosphorylating Tyk2 and STATs (19). PIAS interact with STATs and inhibit their transcriptional activity (20). In addition, the cellular abundance of available STAT proteins can be diminished through specific proteasomal degradation mechanisms (21). Upregulation or activation of any of these proteins or mechanisms leads to a decrease in STAT phosphorylation and ISG expression, thus creating a controlled immune response to infection. As such, viruses have evolved strategies to hijack these innate regulatory features. For instance, RSV (Respiratory Syncytial Virus) upregulates *SOCS1* and *SOCS3*, which in turn inhibits the IFN antiviral response (22), and Dengue virus (DENV) increases USP18, resulting in attenuated IFN-α-induced JAK/STAT signalling (23).

Our previous findings demonstrated that expression of MERS-CoV-nsp2 and SARS-CoV-1-nsp14 in A549 cells led to basal STAT1 & STAT2 phosphorylation, without any exogenous IFN treatment (13). These findings suggested that MERS-CoV-nsp2 and SARS-CoV-1-nsp14 induces cytokine production, that may trigger paracrine/autocrine signalling, thus inducing basal pSTAT1 and pSTAT2. A diverse array of cytokines can activate the JAK/STAT signalling pathway. STAT1 and STAT2 activation is majorly associated with IFNs and previous studies have shown that MERS-CoV and SARS-CoV-1 patients produce numerous cytokines, including IFNs. Indeed, cytokines were significantly higher in patients with severe infection, compared to those with milder infection (24). Given this, we hypothesised MERS-CoV-nsp2 and SARS-CoV-1-nsp14 may trigger the production of IFNs, contributing to basal STAT1 & STAT2 phosphorylation and associated negative regulators, which subsequently inhibit antiviral JAK/STAT signalling. In testing this hypothesis, we sought to elucidate the mechanistic immune evasion roles of MERS-CoV and SARS-CoV-1, towards the identification of novel therapeutic targets for existing and possible future emergent CoVs.

## 2 Materials and methods

### 2.1 Cell culture

The alveolar basal epithelial A549 cell line (a kind gift from Dr Kim Roberts, Trinity College Dublin), the bronchial epithelial BEAS 2b cell line (a kind gift from Prof Ultan Power, Queen’s University Belfast), were cultured in DMEM containing 10% FBS and 1% Penicillin/Streptomycin at 37 °C in a humidified incubator with 5% CO_2_.

### 2.2 Transfection

Cells were transfected with 1 μg DNA constructs encoding HA-tagged MERS-CoV-nsp2, MERS-CoV-nsp5, SARS-CoV-1-nsp14 or the EV control pCAGGS (kind gifts from Prof Matthew Frieman, University of Maryland), using Lipofectamine 2000 (Invitrogen, San Diego, CA, USA) at a ratio of 2 µL lipofectamine: 1 µg of DNA in 2 ml of medium per well (6 well plate).

### 2.3 Treatment of cells

After seeding and transfection cells were treated with different cytokines or stimulus (Human IFN-α2, 10 or 100 ng/ml, Sigma-Aldrich; Human IFN-β, 10 or 100 ng/ml, Proteintech; Human IFN-λ1, 10 or 100 ng/ml, Proteintech; Human IFN-λ3, 10 or 100 ng/ml, Proteintech; Brefeldin A, 3 µg/ml, Bio-Sciences; Human IgG, 10 ng/ml, Bio-Techne; Human IFN-λ1 neutralizing Ab, 10 ng/ml, Bio-Techne; Human IFN-λ3 neutralizing Ab, 10 ng/ml, Bio-Techne). The cytokines and stimulus were diluted in serum free DMEM to reach the required concentration.

### 2.4 Immunoblotting

Cells were lysed in RIPA buffer (20 mM Tris-HCl pH 7.4, 150 mM NaCl, 1 mM EDTA pH 8, 1% TRITON-X, and 0.5% SDS) supplemented with 1 mM PMSF, 1 mM Na_3_VO_4_, 5 μg/mL leupeptin, and 1 mM DTT and analysed by immunoblotting using primary antibodies (pSTAT1, 9167, Cell Signalling Technology; pSTAT2 88410, Cell Signalling Technology; STAT1, 9172S, Cell Signalling Technology; STAT2, SC-476, Santa Cruz Biotechnologies; HA, 3724, Cell Signalling Technology; USP18, 4813, Cell Signalling Technology and β-actin A5441-.2ML, Sigma-Aldrich) and HRP-linked secondary anti-mouse or anti-rabbit antibodies (anti-Rabbit, 11859140, Fisher Scientific or anti-Mouse, 10158113, Fisher Scientific) and then visualised using the Bio-rad ChemiDoc MP imaging system. Blots were analysed using Image Lab software (Bio-rad laboratories, New York, NY, USA).

### 2.5 qRT-PCR

Total RNA was extracted from cells using TRIreagent (Sigma, USA) following manufacture instructions. RNA was converted to cDNA using the SensiFAST cDNA Synthesis kit (Bioline, UK). qRT-PCR was performed using SYBR-green (Bio-Rad, USA) following the kit instructions Data analysis was carried out using the 2^−ΔΔct^ method. The relative expression of each result was calculated based on expression of the constitutively expressed housekeeping reference gene ribosomal protein 15 (*RSPS15*). Primer sequences: *MxA* forward GGTGGTGGTCCCCAGTAATG, reverse ACCACGTCCACAACCTTGTCT, *IFN-α4* forward GTGTCTAGATCTGACAACCTCCCAGGGCACA, reverse GTACTGCAGAATCTCTCCTTTCTCCTG, *IFN-β* forward CTAGCACTGGCTGGAATGAGA, reverse CTGACTATGGTCCAGGCACA, IFN-γ forward ACATGAAAATCCTGCAGAGC, reverse TGGGTTGTTGACCTCAAACT, *IFN-λ1* forward AGCTGCAGGCCTTCAAAAAG, reverse TGGGAGTGAATGTGGCTCAG, *RSP15* forward CGGACCAAAGCGATCTCTTC, reverse CGCACTGTACAGCTGCATCA.

### 2.6 Enzyme-linked immunosorbent assay

96-well plates were coated with 100 μL/well of capture antibody diluted in coating buffer and incubated overnight at 2-8 °C. Plates were washed four times with wash buffer and blocked with 100 μL BSA blocking buffer for 1 h at room temperature (RT) on a plate shaker (500 rpm). After four washes, 100 μL of standards and samples were added in duplicate or triplicate and incubated for 2 h at RT with shaking, followed by four washes. Detection antibody (100 μL/well) was added and incubated for 1 h at RT, then washed four times before adding 100 μL/well of avidin-HRP solution for 30 min at RT. Plates were washed five times with brief soaking between washes, and color was developed using 100 μL/well of TMB substrate for 15–30 min at 37 °C. The reaction was stopped with 50 μL/well of phosphoric acid stop solution, and absorbance was read at 450 nm. (IFN-**λ**1/3, DY1598B, R&D)

### 2.7 Statistical analysis

Statistical comparisons between groups were performed using GraphPad Prism statistical analysis software (version 9). Data is represented as the mean ± SEM unless otherwise stated.

## 3 Results

### 3.1 Expression of MERS-CoV-nsp2 and SARS-CoV-1-nsp14 induces IFN-λ mRNA and protein expression in A549 epithelial cells

**Figure 1:**
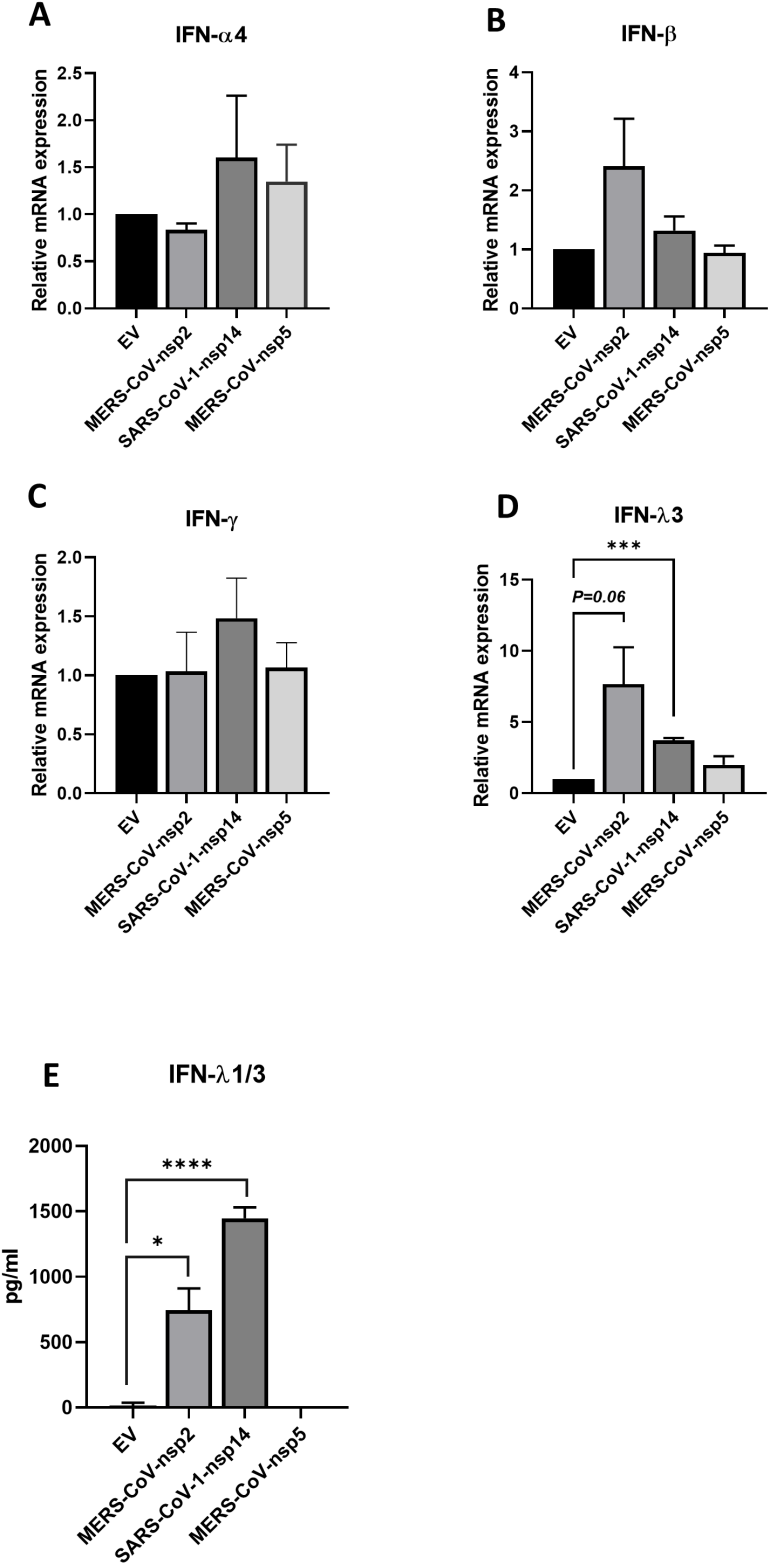
MERS-CoV-nsp2 and SARS-CoV-1-nsp14 increase expression of IFN-λ3 mRNA and protein expression in A549 epithelial cells. A549 cells were transfected with EV control, MERS-CoV-nsp2, SARS-CoV-1-nsp14 or MERS-CoV-nsp5. After 24 h, cells were isolated for total RNA before analysing **(a)** IFN-α4 **(b)** IFN-β **(c)** IFN-γ and **(d)** IFN-λ3 mRNAs by RT-qPCR. Gene expression was normalised to the house-keeping gene *RSP15* and compared to the EV control, which was normalised to 1. After 24 h of MERS-CoV-nsp2, SARS-CoV-1-nsp14 or MERS-CoV-nsp5 transfection, cell supernatants were collected and analysed by ELISA for **(e)** IFN-λ1/3 protein expression from A549 cells. All graphs represent the mean ± SEM of three independent experiments. *p<0.05, ***p<0.001 ****p<0.0001 (student’s t test).

Having previously published that MERS-CoV-nsp2 and SARS-CoV-1-nsp14 expression induced basal phosphorylation of STAT1 and STAT2 in A549 cells (13), we hypothesised that these viral proteins trigger the induction of IFNs, which act in a paracrine/autocrine manner to induce phosphorylation of both STAT1 and STAT2. There are three types of IFNs: type I IFN, type II IFN and type III IFN (25). To initially analyse the effect of MERS-CoV-nsp2 and SARS-CoV-1-nsp14 upon IFN expression, A549 cells were transfected for 24 h with EV, MERS-CoV-nsp2 or SARS-CoV-1-nsp14. MERS-CoV-nsp5 was also used as the control viral protein, that did not induce pSTAT1 & pSTAT2. Then, by qRT-PCR, we measured mRNA levels of the type I IFNs, IFN-α4 and IFN-β; the type II IFN, IFN-γ and the Type III IFN, IFN-λ3. While none of the viral proteins affected mRNA levels of IFN-α4, IFN-β and IFN-γ (**Figures 1A–C**), we observed a statistically significant induction in IFN-λ3 in A549 cells after 24 h SARS-CoV-1-nsp14 expression (**Figure 1D**). Induction of IFN-λ3 upon MERS-CoV-nsp2 expression was near significance (p=0.06) (**Figure 1D**); whereby IFN-λ3 was not impacted by MERS-CoV-nsp5 (**Figure 1D**).

Given that an increase of IFN-λ3 mRNA was observed upon expression of MERS-CoV-nsp2 and SARS-CoV-1-nsp14 (**Figure 1D**), we next analysed cytokine protein levels. A549 cells were transfected with EV control, MERS-CoV-nsp2, SARS-CoV-1-nsp14 or MERS-CoV-nsp5 for 24 h before measuring the secreted IFN-λ3 and IFN-λ1 protein production by ELISA. We found that IFN-λ3, together with IFN-λ1, were significantly evaluated in the cell supernatants from A549 cells transfected with MERS-CoV-nsp2 and SARS-CoV-1-nsp14; while IFN-λ1/3 levels were not altered by MERS-CoV-nsp5 expression (**Figure 1E**). Collectively, these results reveal that MERS-CoV-nsp2 and SARS-CoV-1-nsp14 induced IFN-λ production.

### 3.2 MERS-CoV-nsp2 and SARS-CoV-1-nsp14 expression induces USP18 in A549 epithelial cells

**Figure 2:**
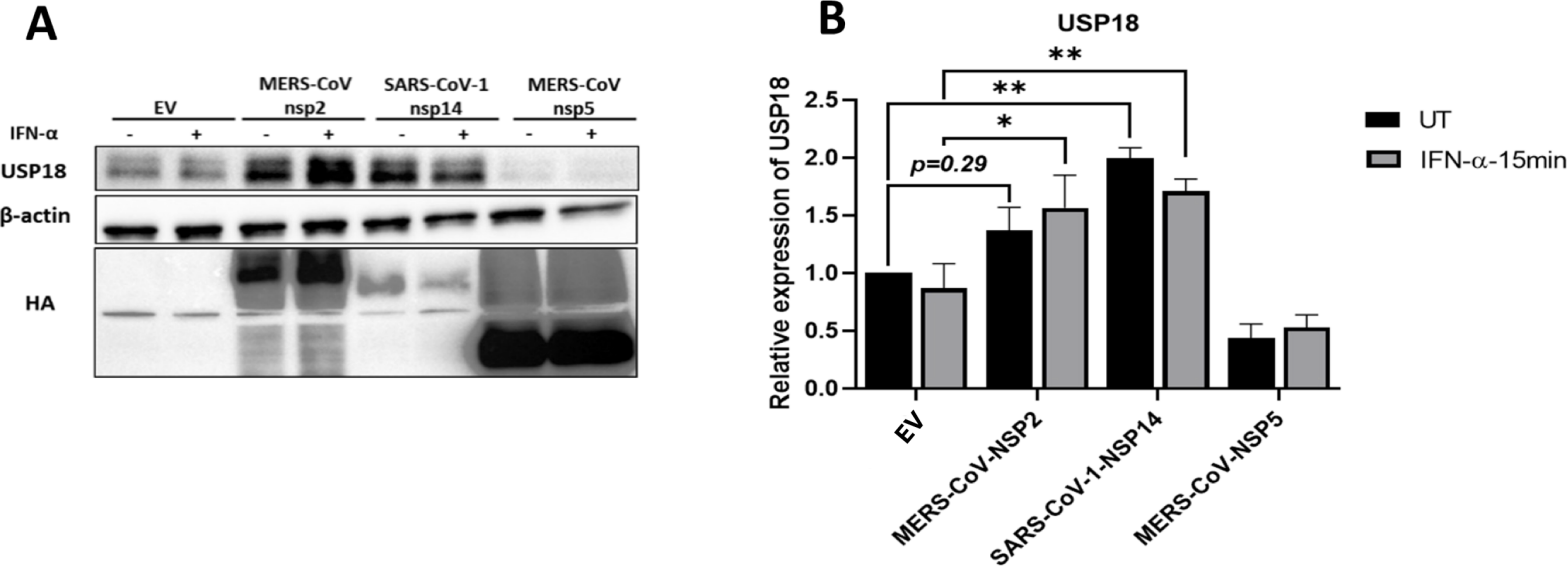
Expression of MERS-CoV-nsp2 and SARS-CoV-1-nsp14 induces USP18 protein expression in A549 epithelial cells. A549 cells were transfected with EV, HA-tagged MERS-CoV-nsp2, SARS-CoV-1-nsp14, or MERS-CoV-nsp5. After 24 h, cells were treated with IFN-α (10 ng/ml) for 15 min. Lysates were generated and subjected to immunoblotting with antibodies for **(a)** USP18 and HA (to confirm expression of HA-tagged MERS-CoV-nsp2, SARS-CoV-1-nsp14 and MERS-CoV-nsp5). Blots were also probed with β-actin antibody. **(b)** Densitometric analysis of **(a)** USP18 was performed using Image Lab software and values were calculated relative to β-actin and compared to the EV transfected UT (untreated) control, which was normalised to 1. All graphs represent the mean ± SEM of three independent experiments. *p<0.05, **p<0.01 (Two-way ANOVA).

USP18 is an inhibitory protein induced by IFN-λ, that can downregulate the type I IFN signalling pathway by disrupting the IFNAR and JAK complex (26). Having observed IFN-λ1/3 induction upon MERS-CoV-nsp2 and SARS-CoV-1-nsp14 (**Figure 1**), we hypothesised that IFN-λ might also increase USP18 expression, possibly explaining why our previously observed reduction in IFN-α-induced ISGs (13). To investigate this, A549 cells were transfected with EV control, MERS-CoV-nsp2, SARS-CoV-1-nsp14 or MERS-CoV-nsp5 for 24 h, prior to stimulation with IFN-α for 15 min, before USP18 protein was analysed by western blotting. Indeed, while western blotting visually indicated that USP18 increased upon expression in MERS-CoV-nsp2 and SARS-CoV-1-nsp14 expressing cells (**Figure 2A**), densitometric analysis revealed a statistically significant increase in USP18 in MERS-CoV-nsp2 expressing cells, with IFN-α treatment, and SARS-CoV-1-nsp14 expressing cells, with or without IFN-α treatment (**Figure 2B**). But USP18 did not increase upon MERS-CoV-nsp5 expression (**Figures 2A, B**).

### 3.3 MERS-CoV-nsp2 and SARS-CoV-1-nsp14 expression does not induce IFN-λ1/3 protein expression, but upregulates USP18 expression in BEAS 2b cells

**Figure 3:**
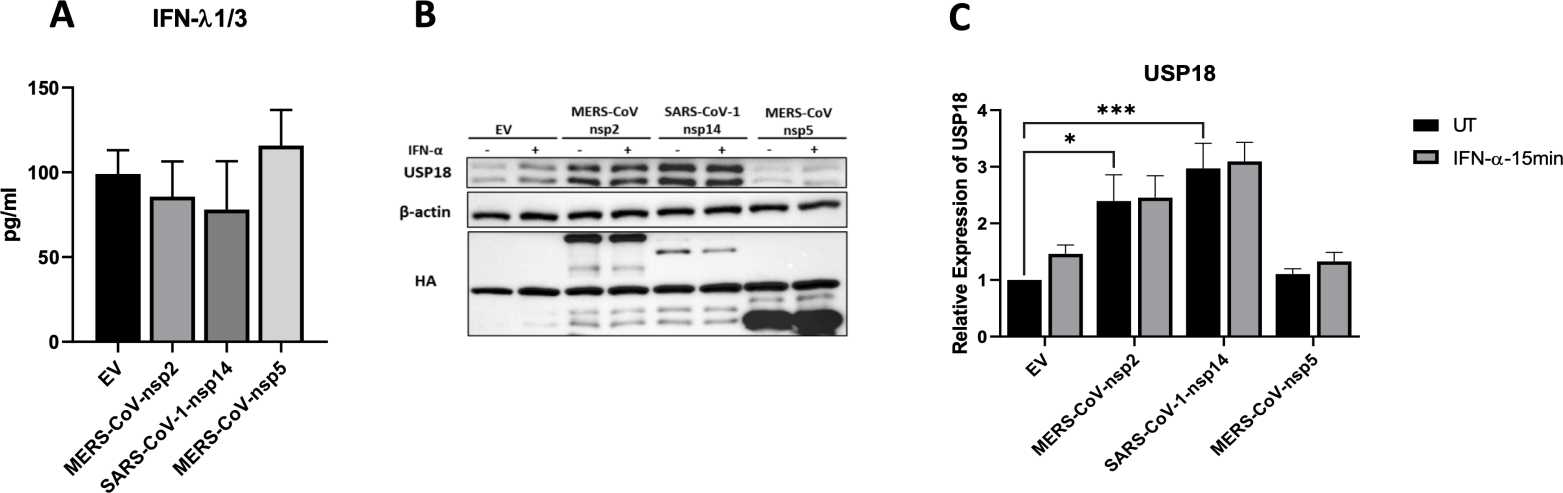
MERS-CoV-nsp2 and SARS-CoV-1-nsp14 expression does not induce IFN-λ1/3 protein expression, but upregulates USP18 expression in BEAS 2b cells. **(a)** BEAS 2b cells were transfected with EV control, MERS-CoV-nsp2, SARS-CoV-1-nsp14 or MERS-CoV-nsp5. After 24 h, cell supernatant was collected and analysed by ELISA for IFN-λ1/3 expression in BEAS 2b cells. BEAS 2b cells were transfected with EV, MERS-CoV-nsp2, SARS-CoV-1-nsp14, or MERS-CoV-nsp5. After 24 h, cells were treated with IFN-α (10ng/ml) for 15 min. Lysates were generated and subjected to immunoblotting with antibodies for **(b)** USP18 and HA. All blots were also probed with β-actin antibody. Densitometric analysis of **(c)** USP18 was performed using Image Lab software and values were calculated relative to β-actin and compared to the EV transfected UT (untreated) control, which was normalised to 1. All graphs represent the mean ± SEM of three independent experiments. *p<0.05, ***p<0.001 (student’s t test, Two-way ANOVA).

Since, in our previous study, MERS-CoV-nsp2 and SARS-CoV-1-nsp14 induced basal phosphorylation of STAT1 & STAT2 in A549 cells, but not BEAS 2b cells (13), we hypothesised that IFN-λ1/3 may be secreted by A549 cells but not by BEAS 2b cells, thereby acting in a paracrine/autocrine manner to induce pSTAT1 & 2 in A549, but not BEAS 2b cells. To investigate this, we then measured the IFN-λ1/3 cytokine production levels in BEAS 2b cells. BEAS 2b cells were transfected with EV control, MERS-CoV-nsp2, SARS-CoV-1-nsp14 or MERS-CoV-nsp5 for 24 h before measuring the IFN-λ1/3 cytokine production by ELISA. No induction of IFN-λ1/3 was observed upon transfection of any of the viral genes, compared to the EV control (**Figure 3A**), suggesting that the transfection of MERS-CoV-nsp2 and SARS-CoV-1-nsp14 induces IFN-λ production only in A549, and not BEAS 2b cells.

Having seen USP18 was induced by MERS-CoV-nsp2 and SARS-CoV-1-nsp14 expression in alveolar A549 cells, we next measured USP18 expression in bronchial BEAS 2b cells. As well as being induced by IFN-λ, USP18 serves as a distinctive inhibitor of type I IFN signalling pathway (23). To check if USP18 is induced after MERS-CoV-nsp2 and SARS-CoV-1-nsp14 expression in BEAS 2b cells, BEAS 2b cells were transfected with EV control, MERS-CoV-nsp2, SARS-CoV-1-nsp14 or MERS-CoV-nsp5 for 24 h, prior to stimulation with and without IFN-α for 15 min. Expression of USP18 protein was determined by western blotting. The USP18 protein expression was significantly induced by MERS-CoV-nsp2 and SARS-CoV-1-nsp14 (**Figures 3B, C**). Since we previously observed no increase in IFN-λ expression by MERS-CoV-nsp2 and SARS-CoV-1-nsp14 in BEAS 2b cells (**Figure 3A**), induction of USP18 may be independent of IFN-λ production in BEAS 2b cells.

### 3.4 Blocking cytokine secretion inhibits MERS-CoV-nsp2 and SARS-CoV-1-nsp14 induced pSTAT1 and IFN-λ1/3 in A549 epithelial cells

**Figure 4:**
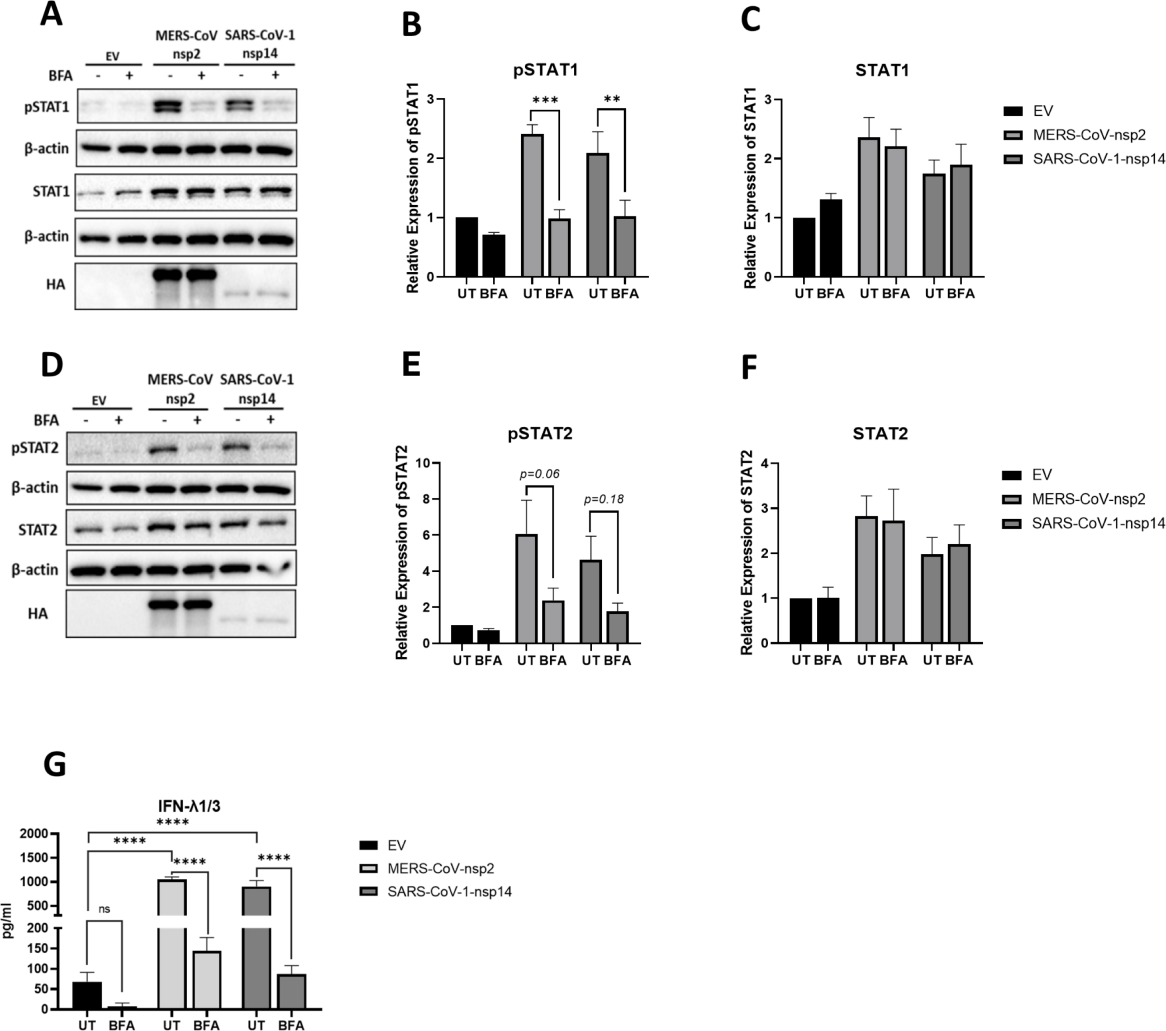
Brefeldin A decreases pSTAT1 and IFN-λ1/3 induction in MERS-CoV-nsp2 or SARS-CoV-1-nsp14 expressing A549 epithelial cells. A549 cells were transfected with EV control, HA-tagged MERS-CoV-nsp2 or SARS-CoV-1-nsp14. After 24 h, cells were treated with or without 3 ug/ml BFA for 16 h Lysates were generated and subjected to immunoblotting with antibodies for **(a)** pSTAT1, STAT1 and HA, **(d)** pSTAT2, STAT2 and HA. All blots were also probed with β-actin antibody. Densitometric analysis of **(b)** pSTAT1, **(c)** STAT1, **(e)** pSTAT2 and **(f)** STAT2 was performed using Image Lab software and values were calculated relative to β-actin and compared to the EV transfected UT (untreated) control, which was normalised to 1. **(g)** Cell supernatants were also collected and subjected into ELISA for IFN-λ1/3. All graphs represent the mean ± SEM of three independent experiments. **p<0.01, ***p<0.001, ****p<0.0001 (Two-way ANOVA).

Next, to investigate if MERS-CoV-nsp2 and SARS-CoV-1-nsp14 expression triggered the production of cytokines, which acted in a paracrine/autocrine fashion to induce basal pSTAT1/pSTAT2 (13) and USP18 (**Figure 2**), we blocked cytokine secretion using Brefeldin A (BFA) (27). A549 cells were transfected with EV, MERS-CoV-nsp2 or SARS-CoV-1-nsp14 for 24 h, before being treated with/without BFA for 16 h, before total STAT1/2 and pSTAT1/pSTAT2 were measured by western blotting. BFA treatment statistically decreased basal phosphorylation of pSTAT1 induced by MERS-CoV-nsp2 and SARS-CoV-1-nsp14 (**Figures 4A, B**), while STAT1 protein was unaffected (**Figure 4C**). While pSTAT2 was visually reduced by BFA (**Figure 4D**), densitometric analysis revealed it was not statistically significant (**Figure 4E**). STAT2 protein levels were not affected by BFA (**Figure 4F**). Together these results indicate that MERS-CoV-nsp2 and SARS-CoV-1-nsp14 expression induce cytokine secretion, that acts in a paracrine/autocrine fashion to induce significant phosphorylation of STAT1.

Next, in a bid to determine if IFN-λ1/3 was responsible for the paracrine/autocrine induction of pSTAT1, we analyzed if their expression was suppressed by BFA. A549 cells were transfected with EV, MERS-CoV-nsp2 or SARS-CoV-1-nsp14 for 24 h, followed with/without BFA treatment for 16 h. Indeed, analysis of the cell supernatants by ELISA revealed that BFA statistically reduced MERS-CoV-nsp2- and SARS-CoV-1-nsp14-induced IFN-λ1/3 (**Figure 4G**), suggesting their possible role in basal STAT1 phosphorylation.

### 3.5 IFN-λ1 & IFN-λ3 neutralizing antibodies block MERS-CoV-nsp2 and SARS-CoV-1-nsp14 expression induced pSTAT1 and USP18 in A549 epithelial cells

**Figure 5:**
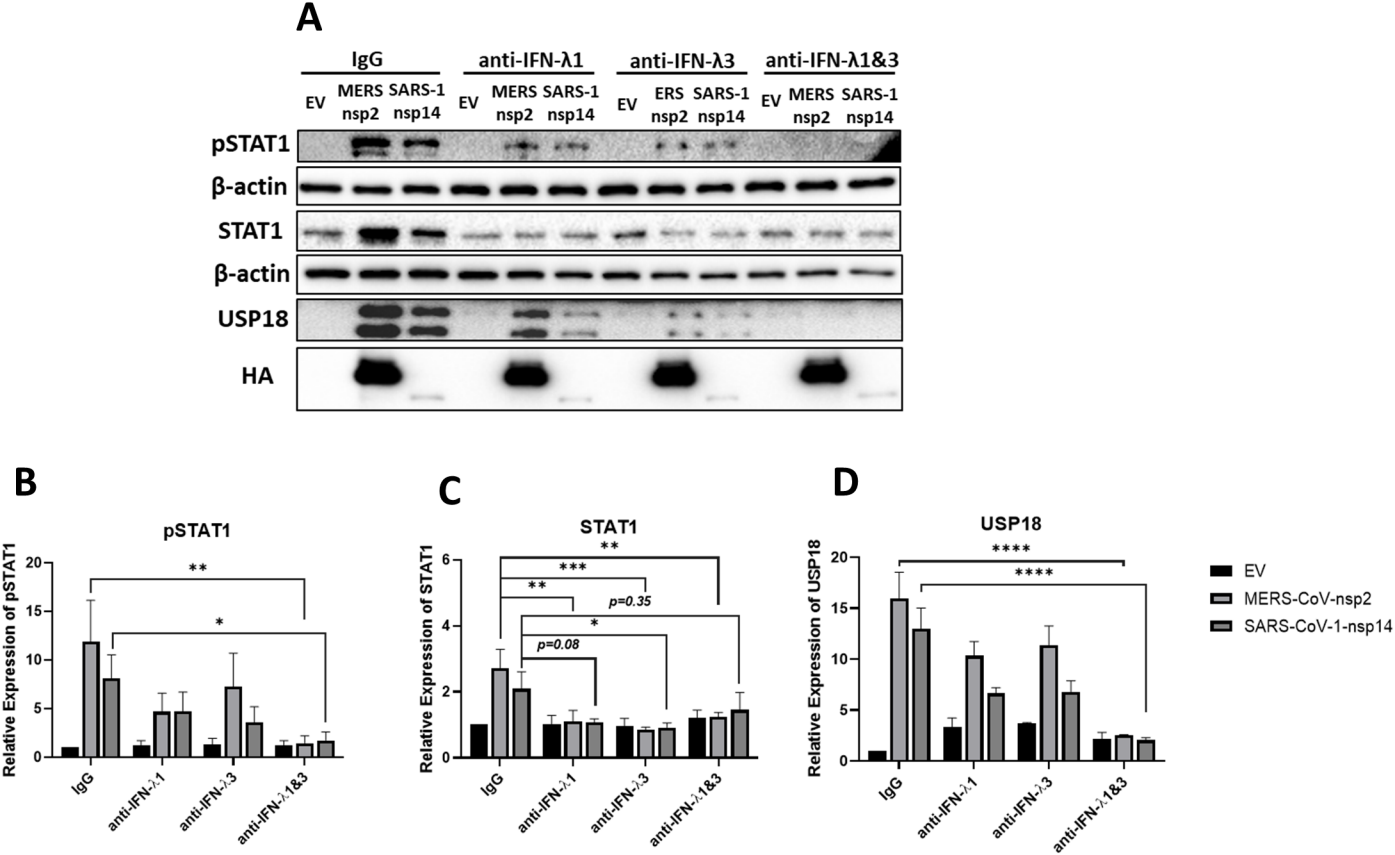
IFN-λ1 & IFN-λ3 neutralizing antibodies reduce pSTAT1 and USP18 in MERS-CoV-nsp2 and SARS-CoV-1-nsp14 expressing A549 cells. A549 cells were transfected with EV control, HA-tagged MERS-CoV-nsp2 or SARS-CoV-1-nsp14. After 24 h, cells were treated with 100 ng/ml IgG, IFN-λ1, IFN-λ3 or IFN-λ1 & 3 neutralizing antibodies for 20 h Lysates were generated and subjected to immunoblotting with antibodies for **(a)** pSTAT1, USP18 and HA. All blots were also probed with β-actin antibody. Densitometric analysis of **(b)** pSTAT1, **(c)** STAT1 and **(d)** USP18 was performed using Image Lab software and values were calculated relative to β-actin and compared to the EV transfected IgG treated control, which was normalised to 1. All graphs represent the mean ± SEM of three independent experiments. *p<0.05, **p<0.01, ***p<0.001, ****p<0.0001 (Two-way ANOVA).

To determine if MERS-CoV-nsp2- and SARS-CoV-1-nsp14-induced pSTAT1 and USP18 were specifically due to secreted IFN-λ1/3, neutralizing antibodies for IFN-λ1 and IFN-λ3 were administered. A549 cells were transfected with EV control, MERS-CoV-nsp2 or SARS-CoV-1-nsp14 for 24 h, followed with IgG control, IFN-λ1, IFN-λ3, or IFN-λ1 and IFN-λ3 neutralizing antibody treatment for 20 h. We observed that while pSTAT1 was not reduced by individual IFN-λ1 or IFN-λ3 blocking antibodies, it was significantly abrogated when the two blocking antibodies were combined (**Figures 5A, B**). Also, the increase in total STAT1 mediated by MERS-CoV-nsp2 and SARS-CoV-1-nsp14 was reduced by IFN-λ3 neutralizing antibody alone. IFN-λ1 blockade significantly suppressed the MERS-CoV-nsp2-induced STAT1, while the reduction of SARS-CoV-1-nsp14-induced STAT1 was near significance (p=0.08). Added together IFN-λ1 and IFN-λ3 neutralizing antibodies reduced MERS-CoV-nsp2-induced STAT1, while the reduction of SARS-CoV-nsp14-induced STAT1 was not significant (**Figures 5A, C**). While individual neutralizing antibodies did not reduce USP18, treatment with both IFN-λ1 and IFN-λ3 blocking antibodies significantly reduced MERS-CoV-nsp2- and SARS-CoV-1-nsp14-induced USP18 (**Figures 5A, D**). Collectively, these results indicate that IFN-λ1/3 produced by MERS-CoV-nsp2 and SARS-CoV-1-nsp14 expressing A549 cells, may activate STAT1, while also increasing the inhibitory protein, USP18.

### 3.6 IFN-λ1 & IFN-λ3 neutralization restores IFN-α responsiveness of MERS-CoV-nsp2 and SARS-CoV-1-nsp14 expressing A549 cells

**Figure 6:**
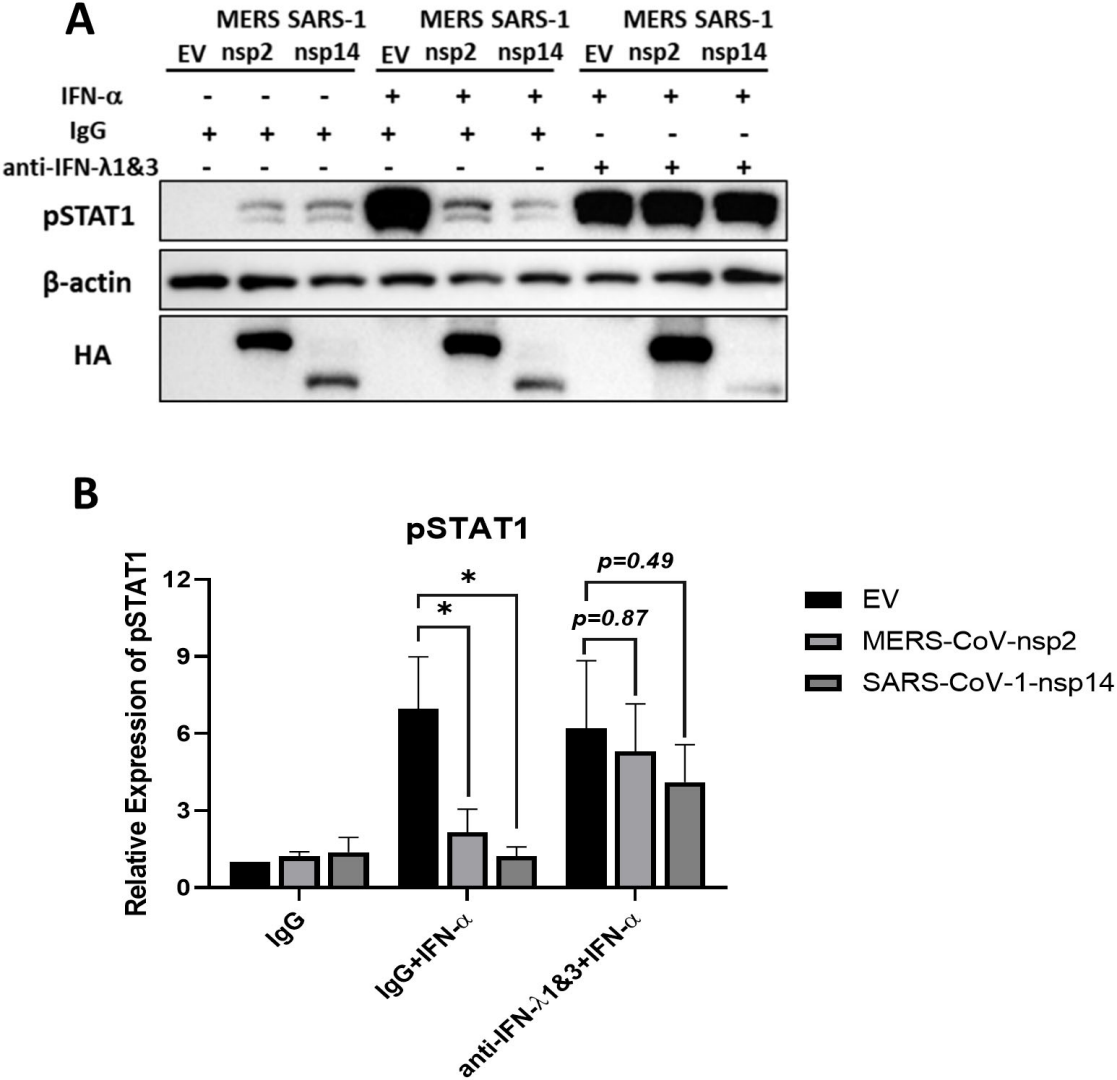
IFN-λ1 & IFN-λ3 neutralizing antibodies restore IFN-α responsiveness in MERS-CoV-nsp2 and SARS-CoV-1-nsp14 expressing A549 cells. A549 cells were transfected with EV control, HA-tagged MERS-CoV-nsp2 or SARS-CoV-1-nsp14. After 24 h, cells were treated with 100 ng/ml IgG or IFN-λ1 and IFN-λ3 neutralizing antibodies for 20 h and followed with IFN-α treatment (10 ng/ml) for 15 min. Lysates were generated and subjected to immunoblotting with antibodies for **(a)** pSTAT1 and HA. All blots were also probed with β-actin antibody. Densitometric analysis of **(b)** pSTAT1 was performed using Image Lab software and values were calculated relative to β-actin and compared to the EV transfected IgG treated IFN-α untreated control, which was normalised to 1. All graphs represent the mean ± SEM of three independent experiments. *p<0.05 (Two-way ANOVA).

Having previously shown that MERS-CoV-nsp2 and SARS-CoV-1-nsp14 expression blocked IFN-α-mediated pSTAT1 and ISGs (13), we wondered if the induction of IFN-λ1/3, its paracrine/autocrine effect and its induction of USP18, might be responsible for this inhibition. To investigate this, A549 cells were transfected with EV, MERS-CoV-nsp2 or SARS-CoV-1-nsp14 for 24 h, followed with IgG or IFN-λ1 & IFN-λ3 neutralizing antibody treatment. After 20 h cells were further treated with IFN-α for 15 min. We found that when cells were treated with IgG control, both MERS-CoV-nsp2 and SARS-CoV-1-nsp14 expression significantly reduced IFN-α-mediated pSTAT1. However, treatment with IFN-λ1 and IFN-λ3 neutralizing antibodies restored IFN-α-induced pSTAT1 (**Figures 6A, B**). These data indicate that IFN-λ1/3, secreted upon MERS-CoV-nsp2 and SARS-CoV-1-nsp14 expression, reduced IFN-α responsiveness of A549 cells.

### 3.7 Silencing USP18 in A549 cells expressing MERS-CoV-nsp2 and SARS-CoV-1-nsp14, restores IFN-α-mediated pSTAT1 and IFN-α-mediated *MxA* induction in SARS-CoV-1-nsp14 expressing cells

**Figure 7:**
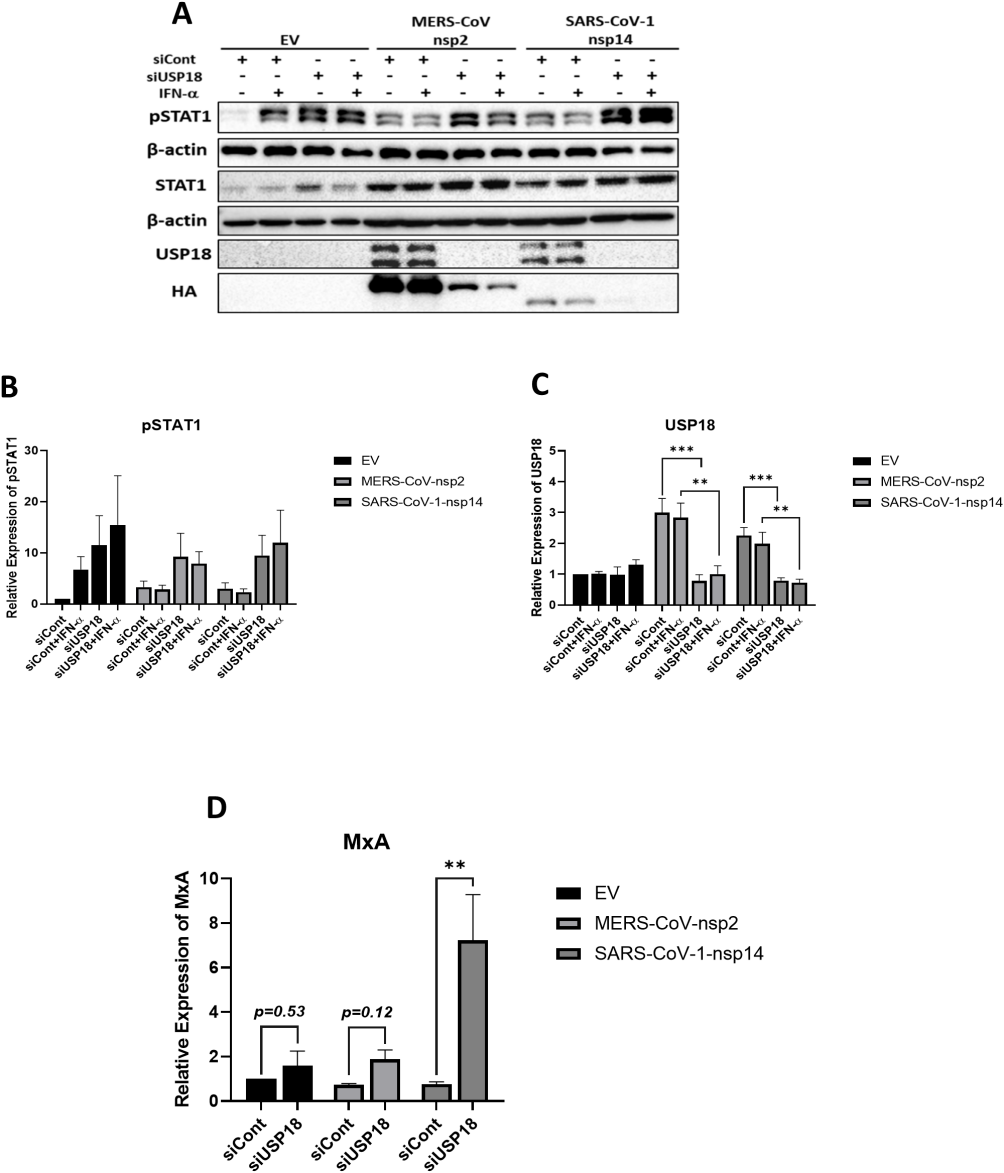
Silencing USP18 restores IFN-α-mediated STAT1 phosphorylation in A549 cells expressing MERS-CoV-nsp2 and SARS-CoV-1-nsp14 and *MxA* in SARS-CoV-1-nsp14 expressing A549 cells. A549 cells were transfected with EV control, HA-tagged MERS-CoV-nsp2 or SARS-CoV-1-nsp14. After 24h, cells were transfected with 1 ug control siRNA or USP18 siRNA for 24 h followed with IFN-α treatment (10 ng/ml) for **(a–c)** 15 min or (d) 4h. **(a–c)** Lysates were generated and subjected to immunoblotting with antibodies for **(a)** pSTAT1, STAT1, USP18 and HA. All blots were also probed with β-actin antibody. Densitometric analysis of **(b)** pSTAT1 and **(c)** USP18 was performed using Image Lab software and values were calculated relative to β-actin and compared to the control siRNA transfected IFN-α untreated EV control, which was normalised to 1. **(d)** *MxA* mRNA in A549 cells was measured by RT-qPCR. Gene expression was normalised to house-keeping gene *RSP15* and IFN-α treated samples were compared to the control siRNA transfected, IFN-α treated EV control, which was normalised to 1. All graphs represent the mean ± SEM of three independent experiments. **p<0.01, ***p<0.001 (Two-way ANOVA).

Having observed that MERS-CoV-nsp2 and SARS-CoV-1-nsp14 expression induced IFN-λ1/3, we wondered if USP18, which is known to diminish IFN-α signalling (26), might be responsible for reduced IFN-α responsiveness. To investigate this, USP18 siRNA knock-down experiments were conducted. USP18 expression was confirmed to decrease significantly after USP18 siRNA transfection (**Figures 7A, C**). In control siRNA-transfected cells, MERS-CoV-nsp2 or SARS-CoV-1-nsp14 transfection attenuated IFN-α-mediated pSTAT1, however, pSTAT1 was restored upon USP18 siRNA knock-down (**Figures 7A, B**). We next investigated if downstream ISG induction was consequently restored. We found that *MxA* mRNA expression was significantly increased after knock-down of USP18 in A549 cells transfected with SARS-CoV-1-nsp14; *MxA* induction was not statistically restored in cells transfected with MERS-CoV-nsp2 (**Figure 7D**). Combined our results indicate that IFN-α unresponsiveness in A549 cells expressing MERS-CoV-nsp2 and SARS-CoV-1-nsp14 is, at least in part, regulated by USP18.

### 3.8 Silencing USP18 in BEAS 2b cells expressing MERS-CoV-nsp2 and SARS-CoV-1-nsp14, restores IFN-α-mediated pSTAT1 and IFN-α-mediated *MxA* induction in MERS-CoV-nsp2 and SARS-CoV-1-nsp14 expressing cells

**Figure 8:**
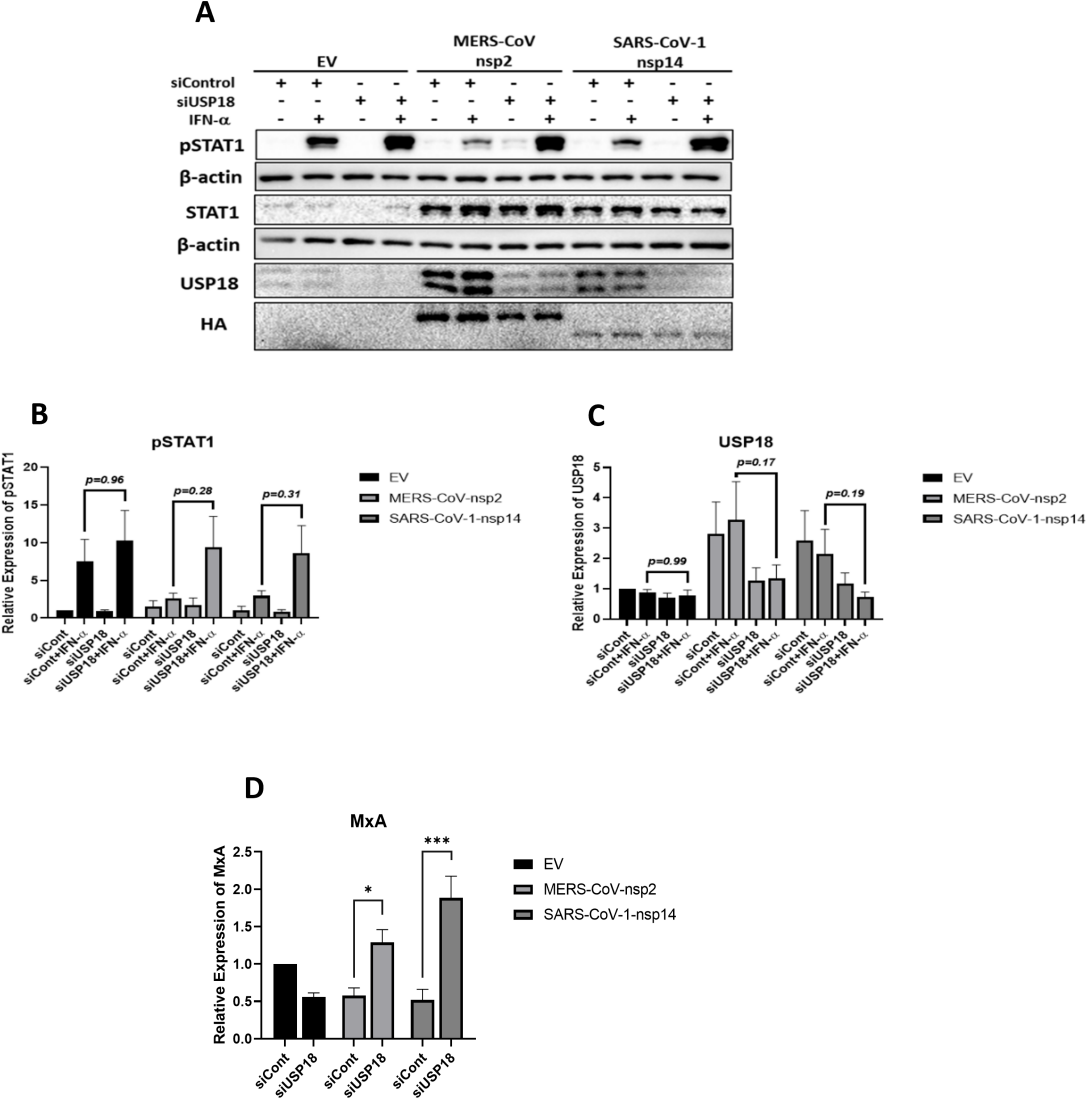
Silencing USP18 restores IFN-α-mediated STAT1 phosphorylation and *MxA* in MERS-CoV-nsp2 and SARS-CoV-1-nsp14 expressing BEAS 2b cells. BEAS 2b cells were transfected with EV control, HA-tagged MERS-CoV-nsp2 or SARS-CoV-1-nsp14. After 24 h, cells were transfected with 1 ug control siRNA or USP18 siRNA for 24 h followed with IFN-α treatment (10 ng/ml) for **(a–c)** 15 min or **(d)** 4h. Lysates were subjected to immunoblotting with antibodies for **(a)** pSTAT1, STAT1, USP18 and HA. All blots were also probed with β-actin antibody. Densitometric analysis of **(b)** pSTAT1 and **(c)** USP18 was performed using Image Lab software and values were calculated relative to β-actin and compared to the control siRNA transfected IFN-α untreated EV control, which was normalised to 1. **(d)** *MxA* mRNA in BEAS 2b cells was measured by RT-qPCR. Gene expression was normalised to house-keeping gene *RSP15* and IFN-α treated samples were compared to the control siRNA transfected, IFN-α treated EV control, which was normalised to 1. All graphs represent the mean ± SEM of three independent experiments. *p<0.05, ***p<0.001 (Two-way ANOVA).

Since we also observed induced USP18 (**Figure 3**) and inhibited STATs phosphorylation upon the expression of MERS-CoV-nsp2 and SARS-CoV-1-nsp14 in BEAS 2b bronchial epithelial cells (13), we hypothesised that USP18 could be responsible for the unresponsiveness to IFN-α in BEAS 2b cells. To explore this, we next performed the same experiments in BEAS 2b cells to investigate the role of USP18 in IFN-α unresponsiveness. Transfection of USP18 siRNA resulted in a visibly decreased level of USP18 observed from blots, although this decrease was not statistically significant. (**Figures 8A, C**). Similarly to A549 cells, knock-down the expression of USP18 restored IFN-α-induced STAT1 phosphorylation by approximately 4-fold and 3-fold in BEAS 2b cells expressing MERS-CoV-nsp2 and SARS-CoV-1-nsp14, respectively (**Figures 8A, B**). Although the densitometry analysis is not statistically significant, the enhanced STAT1 phosphorylation levels after USP18 knock-down was clearly visible from blots. We next measured downstream ISG induction to determine if it was consequently restored. As shown in **Figure 8D**, *MxA* mRNA expression was significantly increased after knock-down of USP18 expression in BEAS 2b cells expressing MERS-CoV-nsp2 and SARS-CoV-1-nsp14. Combined with our previous results showing restored STAT1 phosphorylation after USP18 knock-down (**Figure 7**), these data together suggest that IFN-α unresponsiveness in both A549 and BEAS 2b cells depends on USP18.

### 3.9 Pretreatment of A549 epithelial cells with IFN-λ1 or IFN-λ3 attenuates IFN-α-mediated STAT1 phosphorylation

**Figure 9:**
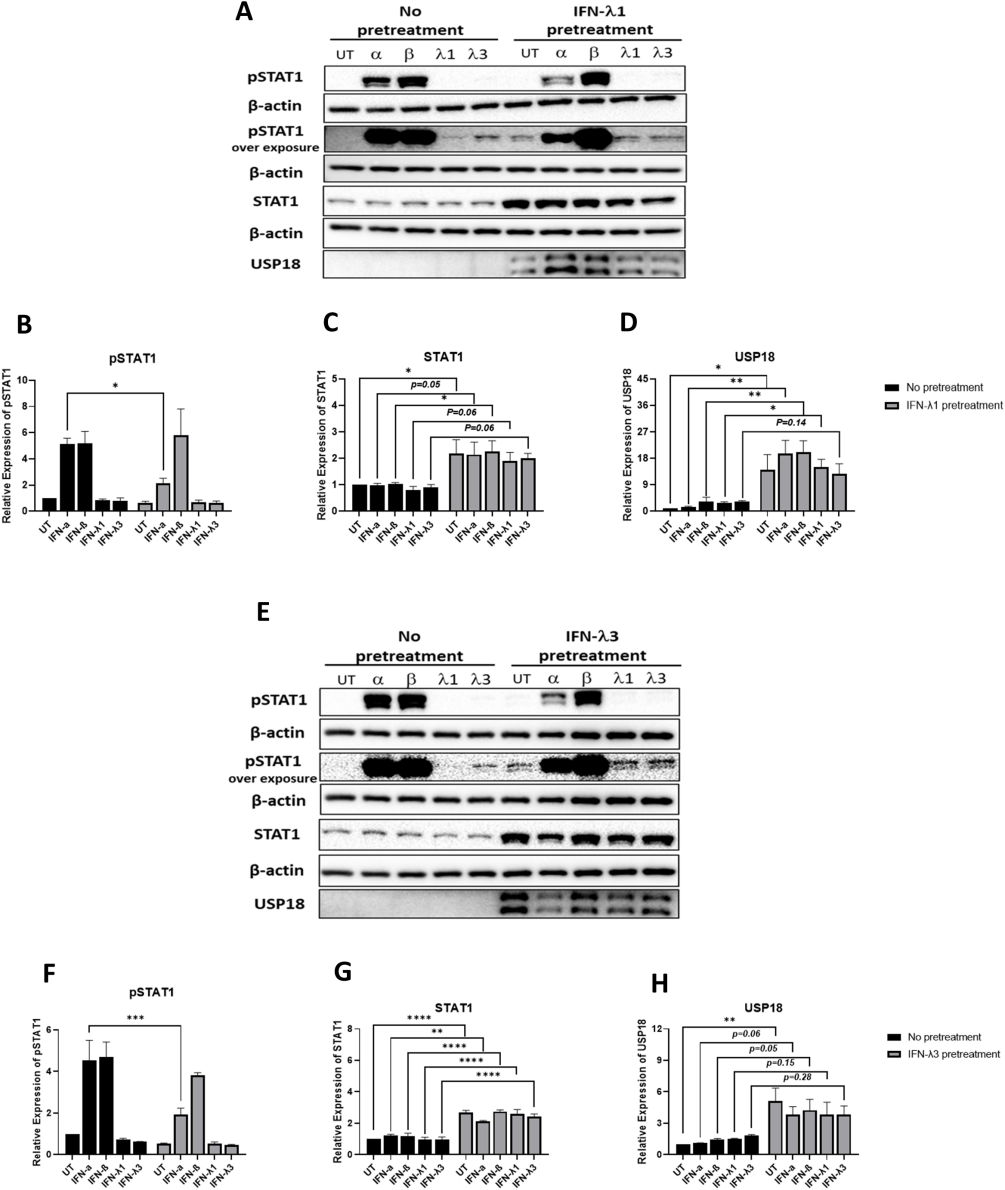
A549 epithelial cells pretreated with IFN-λ1 or IFN-λ3 show weaker STAT1 phosphorylation in response to IFN-α treatment. A549 cells were pretreated with 10 ng/mL IFN-λ1 or IFN-λ3 for 24 h and then treated with 10 ng/ml of IFN-α, IFN-β, IFN-λ1 or IFN-λ3 for another 15 min. Lysates were generated and subjected to immunoblotting with antibodies for **(a)** pSTAT1, STAT1 and USP18 in A549 cells pretreated with IFN-λ1 and **(e)** pSTAT1, STAT1 and USP18 in in A549 cells pretreated with IFN-λ3. All blots were also probed with β-actin antibody. Densitometric analysis of **(b)** and **(f)** pSTAT1, **(c)** and **(g)** STAT1 and **(d)** and **(h)** USP18 was performed using Image Lab software and values were calculated relative to β-actin and compared to the no pretreated and untreated (UT) control, which was normalised to 1. All graphs represent the mean ± SEM of three independent experiments. *p < 0.05, **p < 0.01, ***p < 0.001, ****p < 0.0001 (Two-way AVONA).

Since IFN-λ induces USP18 (28, 29), we wondered if this was responsible for the reduced responsiveness to IFN-α we observed in the presence of MERS-CoV-nsp2 or SARS-CoV-1-nsp14 in A549 epithelial cells (**Figure 7**). To investigate this, A549 cells were pretreated with 10 ng/mL IFN-λ1 for 24 h, then 10 ng/ml of IFN-α, IFN-β, IFN-λ1 or IFN-λ3 for another 15 min. We found that pretreatment with IFN-λ1 resulted in a statistically significant induction of USP18, in all treatments except IFN-λ1 24h plus IFN-λ1 15min (**Figures 9A, D**). Pretreatment with IFN-λ1 also resulted in a statistically significant or near significant (P = 0.05-0.06) induction of STAT1 expression (**Figures 9A, C**). Cells pretreated with IFN-λ1 showed much weaker IFN-α-induced pSTAT1 after treatment with exogenous IFN-α, than in control cells (without IFN-λ1 pretreatment) (**Figures 9A, B**). However, attenuated responses in STAT1 phosphorylation were not observed after treatment with exogenous IFN-β, IFN-λ1 or IFN-λ3 in IFN-λ1-pretreated cells (**Figures 9A, B**). Having seen that IFN-λ1 pretreatment attenuated response to IFN-α, we next investigated the effect of IFN-λ3 pretreatment. A549 cells were pretreated with 10 ng/mL IFN-λ3 for 24 h followed with 10 ng/ml of IFN-α, IFN-β, IFN-λ1 or IFN-λ3 treatment for another 15 min. Similarly, IFN-λ3 pretreatment also attenuated response to exogenous IFN-α, as demonstrated with reduced pSTAT1 (**Figures 9E, F**). We also observed that STAT1 protein was significantly increased (**Figures 9E, G**). We found that stimulation with IFN-λ3 visually increased USP18, with UT cells showing a statistically significant increase upon IFN-λ1 pretreatment, and IFN-α (p=0.06) and IFN-β (p=0.05) treated cells showing a near significant increase in USP18 upon IFN-λ3 pretreatment (**Figures 7E, H**).

### 3.10 IFN-λ1 or IFN-λ3 pretreatment of A549 epithelial cells reduces IFN-α-mediated *MxA* induction

**Figure 10:**
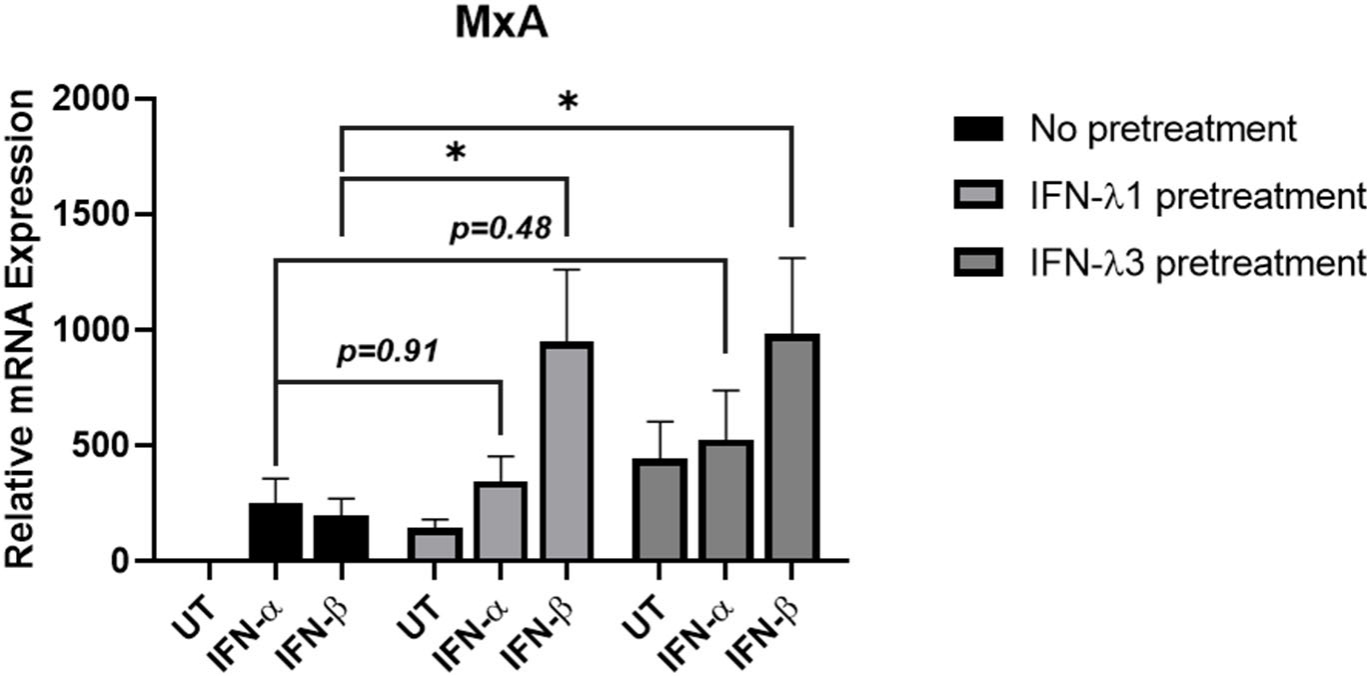
IFN-λ1 and IFN-λ3 pretreatment reduces IFN-α-induced *MxA* induction in A549 cells. A549 cells were pretreated with 10 ng/mL IFN-λ1 or IFN-λ3 for 24 h and then treated with 10 ng/ml of IFN-α or IFN-β for another 4 h, before analysing *MxA* mRNA by RT-qPCR. Gene expression was normalised to house-keeping gene *RSP15* and all treated samples were compared to the untreated (UT) and no pretreatment control, which was normalised to 1. All graphs represent the mean ± SEM of three independent experiments. *p < 0.05 (Two-way ANOVA).

Since IFN-α-mediated pSTAT1 was reduced by IFN-λ1/3 pretreatment we hypothesised that downstream ISG induction would also be attenuated. To investigate this, A549 cells were pretreated with 10 ng/mL IFN-λ1 or IFN-λ3 for 24 h, followed with 10 ng/ml of IFN-α or IFN-β treatment for 4 h. *MxA* mRNA induction was measured by qRT-PCR. We found that *MxA* mRNA remained significantly induced by IFN-β in cells pretreated with IFN-λ1 or IFN-λ3 (**Figure 10**). However, *MxA* was not significantly increased by IFN-α in cells pretreated with IFN-λ1 or IFN-λ3 (**Figure 10**). Combined these data suggest that IFN-λ1/3 induces an inhibitory mechanisim that weakens antiviral signalling responses to IFN-α treatment.

### 3.11 Knock-down of USP18 reverses IFN-λ1- or IFN-λ3-associated inhibition of IFN-α-induced pSTAT1 and *MxA*

**Figure 11:**
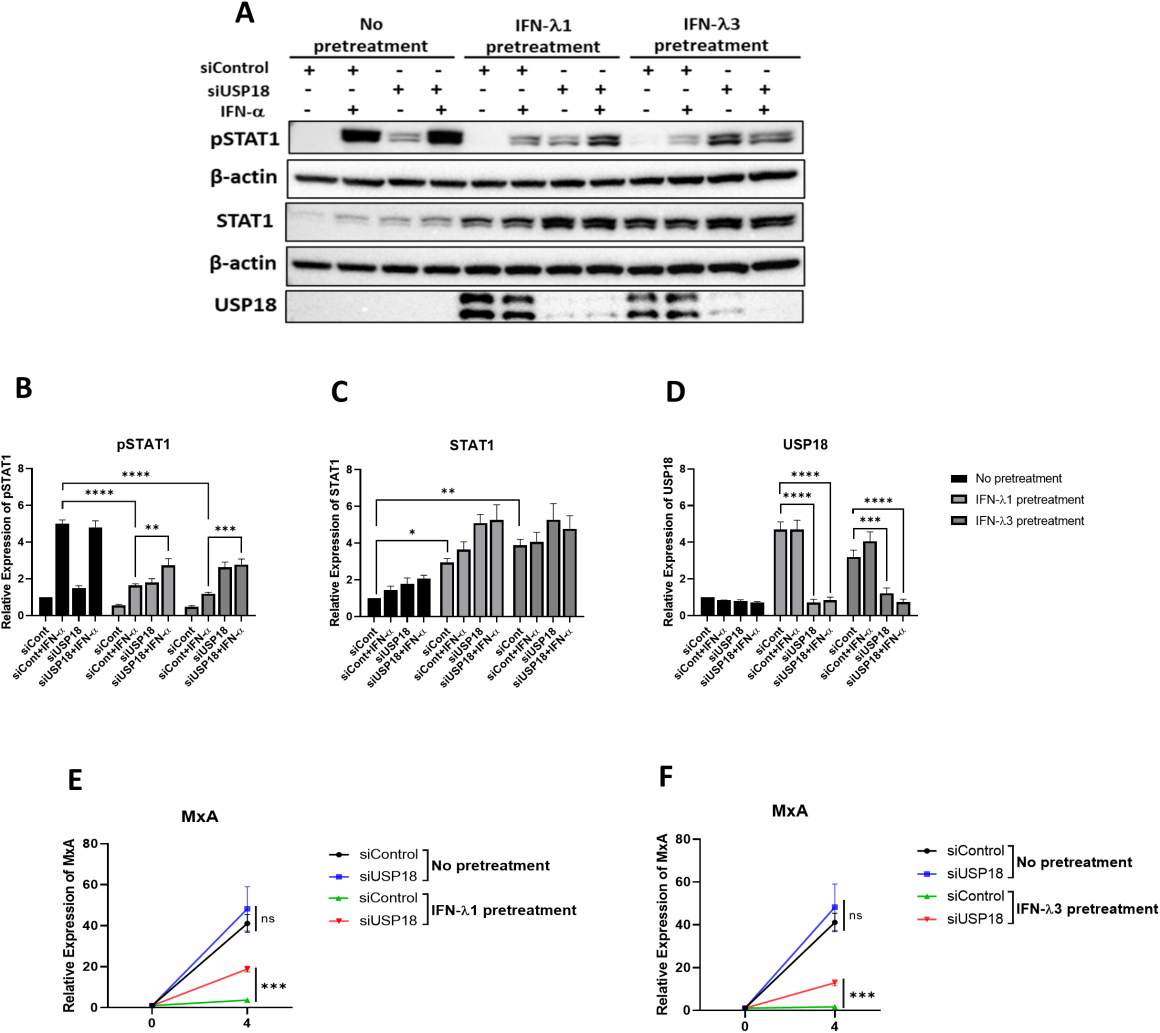
Knock-down of USP18 reverses IFN-λ1- or IFN-λ3-associated inhibition of IFN-α-induced STAT1 phosphorylation and *MxA*. A549 cells were pretreated with 10 ng/ml IFN-λ1 or IFN-λ3 for 24 h, and the indicated siRNAs were introduced by transfection. After 24 h transfection, the cells were treated with 10 ng/ml of IFN-α for **(a–d)** 15 min or (e & f) 4 h **(a–d)** Lysates were generated and subjected to immunoblotting with antibodies for **(a)** pSTAT1, STAT1 and USP18. All blots were also probed with β-actin antibody. Densitometric analysis of **(b)** pSTAT1, **(c)** STAT1 and **(d)** USP18 was performed using Image Lab software and values were calculated relative to β-actin and compared to the no pretreated and untreated (UT) control, which was normalised to 1. **(e, f)** total RNA was extracted before **(e)** *MxA* mRNA in A549 cells pretreated with IFN-λ1 and **(f)** *MxA* mRNA in A549 cells pretreated with IFN-λ3 were measure by qRT-PCR. Gene expression was normalised to house-keeping gene *RSP15* and IFN-α treated samples were compared to the control siRNA transfected, IFN-α untreated control in each group, which was normalised to 1. All graphs represent the mean ± SEM of three independent experiments. *p < 0.05, **p < 0.01, ***p < 0.001, ****p < 0.0001 (Two-way AVONA).

Having observed inhibition of IFN-α-induced pSTAT1 and *MxA* induction upon pretreatment with IFN-λ1/λ3 and induction of USP18, we hypothesised that IFN-λ-induced USP18 was responsible for inhibiting IFN-α signalling responses. To investigate this we conducted USP18 knock-down experiments. A549 cells were pretreated with 10 ng/mL IFN-λ1 or IFN-λ3 for 24 h, followed with transfection of USP18 siRNA for another 24 h. Transfection of USP18 siRNA resulted in a significant reduction in USP18 protein (**Figures 11A, D**). In cells transfected with control siRNA, IFN-λ1 or IFN-λ3 pretreatment significantly reduced IFN-α-mediated pSTAT1, compared to cells without IFN-λ1 or IFN-λ3 pretreatment (**Figures 11A, B**). However, IFN-α-mediated STAT1 phosphorylation was restored in the absence of USP18 pretreated with IFN-λ1 or IFN-λ3 (**Figure 11A, B**). Furthermore, pretreatment of control siRNA cells with IFN-λ1 or IFN-λ3 significantly increased total STAT1 protein (**Figures 11A, C**). Given that IFN-λ1 or IFN-λ3 pretreatment restored pSTAT1 after knock-down of USP18, we next analysed whether downstream ISG induction is also restored by measuring *MxA* mRNA. As shown in **Figures 11E, F**, in cells pretreated with IFN-λ1 or IFN-λ3, IFN-α-induced *MxA* mRNA expression was significantly increased after knock-down of USP18 expression, compared to siRNA control cells pretreated with IFN-λ1 or IFN-λ3. These results indicate that IFN-λ1/IFN-λ3-mediated induction of USP18 is responsible for IFN-α unresponsiveness of A549 epithelial cells.

## 4 Discussion

Several viral infections have been shown to induce the inhibitory protein, USP18, which evades IFN-α JAK/STAT signalling. For example, HCV-infected hepatocytes have been shown to produce type III IFNs and induce USP18, which causes poor response to IFN-α (26). DENV infection has been shown to increase USP18 expression, which impairs the IFN-α JAK/STAT signalling pathway and promotes viral replication (23). Furthermore, trials and retrospective studies with therapeutic IFN-α have shown MERS-CoV and SARS-CoV-1 to have disappointingly weak clinical responses (5–7), suggesting that CoVs encode antagonists that counteract JAK/STAT signalling. Our previous study showed that MERS-CoV-nsp2 and SARS-CoV-1-nsp14 induced a basal level of STAT phosphorylation in A549 cells, while also inhibiting additional IFN-α-mediated pSTAT and downstream ISG induction, in both A549 and BEAS 2b cells (13). In this current study we found that expression of MERS-CoV-nsp2 and SARS-CoV-1-nsp14 in human epithelial A549 cells induced IFN-λ1/3. Using Brefeldin A and IFN-λ1/3 neutralizing antibodies we also found that the basal pSTAT (observed in MERS-CoV-nsp2 and SARS-CoV-1-nsp14 expressing cells), was due to IFN-λ1/3 production, most likely acting in a paracrine/autocrine manner. Stimulation of A549 cells with type III IFN also increased the expression of USP18 and suppressed IFN-α-mediated pSTAT1 and *MxA* induction. Moreover, it was also found that expression of MERS-CoV-nsp2 and SARS-CoV-1-nsp14 in BEAS 2b cells increased the USP18 expression independent of IFN-λ1/3 production, distinguishing the results of BEAS 2b cells from those observed in A549 cells. When USP18 was further silenced in both A549 and BEAS 2b cells, an enhanced STAT1 phosphorylation and *MxA* induction was observed, indicating an increased response to IFN-α. In summary, these findings demonstrated that, MERS-CoV-nsp2 and SARS-CoV-1-nsp14 inhibited IFN-α JAK/STAT signalling pathway through USP18 upregulation.

USP18 is a deubiquitinating enzyme that plays a critical role in regulating various aspects of the immune response (30). Independent of its deubiquitinating activity, it is an important regulator of IFN-α signalling, which blocks the phosphorylation of STAT1 and STAT2 at the level of the receptor-kinase complex (31). It has been reported that USP18 overexpression decreased the responsiveness to exogenous IFN-α (28). In our current study, we demonstrated that MERS-CoV-nsp2 and SARS-CoV-1-nsp14 increased USP18 expression. Silencing of USP18 expression increased the response to IFN-α by restoring pSTAT1 phosphorylation and increasing *MxA* induction in BEAS 2b cells, indicating that MERS-CoV-nsp2- and SARS-CoV-1-nsp14-induced USP18 is responsible for hindering the response to IFN-α. Notably, silencing USP18 restored IFN-α-induced STAT1 phosphorylation in A549 cells expressing MERS-CoV-nsp2 or SARS-CoV-1-nsp14, but significant *MxA* induction was observed only with SARS-CoV-1-nsp14. This indicates that USP18 plays a dominant role in nsp14-mediated ISG suppression, while MERS-CoV-nsp2 may also use USP18-independent mechanisms downstream of STAT phosphorylation, such as blocking STAT nuclear translocation (13), transcriptional complex assembly, or ISG promoter activation. Thus, the contributions of USP18-dependent and -independent pathways vary between viral proteins and epithelial cell type. It has been reported that silencing of USP18 potentiated the anti-DENV activity through promoting the activation of the IFN-α-mediated JAK/STAT signalling pathway, thereby inhibiting viral replication (32). Similar findings have been reported in HBV, whereby silencing of USP18 enhanced the anti-HBV activity of IFN-α (33, 34). Additionally, studies have highlighted the role of USP18 in modulating the anti-HCV type I IFN response, with silencing of USP18 being suggested as a potential therapeutic target for the treatment of HCV infection (35). Collectively, our results, along with prior research, underscore the significance of USP18 as a crucial host factor utilized by viruses to antagonize the IFN-α response.

It is found USP18 is exploited by MERS-CoV and SARS-CoV-1 to suppress IFN-α signalling, but its therapeutic targeting is nuanced. It negatively regulates IFNAR signalling by competing with JAK1 for IFNAR2 binding and serves as the principal deISGylase that removes ISG15 from substrates, maintaining immune homeostasis (36). Systemic USP18 loss prolongs IFN signalling and drives severe immunopathology, as shown in USP18-deficient mice (37), whereas epithelial USP18 silencing enhances IFN responsiveness *in vitro* (35). These findings suggest that therapeutic strategies will require transient or tissue-restricted modulation, selective inhibition of IFNAR regulatory activity while sparing deISGylation, or targeting viral induction of USP18. Collectively, USP18 functions as a marker of Coronavirus (CoVs) immune evasion rather than a straightforward drug target, highlighting the importance of restoring antiviral signalling without disrupting immune balance, an observation consistent with prior studies indicating that USP18 silencing can enhance antiviral IFN responses, including against HCV (35).

In this study, it was found MERS-CoV-nsp2 and SARS-CoV-1-nsp14 particularly induce type III interferons (IFN-λ) in A549 epithelial cells. These interferons signal through a receptor complex composed of IFNLR1 (also known as IL-28Rα) and IL-10Rβ, which are highly expressed on epithelial cells and certain types of myeloid cells (38). Due to this specific expression pattern, the antiviral properties of IFN-λ are particularly prominent at epithelial barriers, such as those in the respiratory, gastrointestinal and reproductive tracts (39–41). Indeed, IFN-λ families are the predominant IFNs produced early during IAV infection by epithelial cells (30). Moreover, in the lungs of mice infected with MERS-CoV, the expression of IFN-λ was significantly elevated, concomitant with an increase in IL-6 (42). During SARS-CoV-1 infection, a marked increase of IFN-λ was detected in human alveolar cells (43). The BEAS 2b cells did not exhibit cytokine induction, and this variance in cytokine response between A549 cells and BEAS 2b cells, following the expression of viral proteins, could be attributed to various factors. A549 and BEAS 2b are derived from different tissues which possess distinct characteristics. A549 cells are alveolar derived human lung carcinoma cells, while BEAS 2b cells are immortalized bronchial epithelial cells, which are closest to normal bronchial epithelium (44). These different origins could result in variations in gene expression, cellular responses, and immune-related cascades. Furthermore, the expression of viral PRRs, such as TLRs, RLRs, and others which are responsible for recognizing viral components and triggering downstream signalling, can differ between cell types. Variations in the expression of these receptors can lead to differences in cytokine induction. Similarly, RSV infection in A549 cells was found to induce higher levels of IFN-λ and NF-κB-inducible proinflammatory cytokines, compared with in BEAS 2b cells. In contrast, BEAS 2b cells expressed higher levels of PRRs and other signalling intermediaries after infection (45). This highlights the distinct response patterns of these cells to the infectious stimulus. The distinct response patterns of A549 and BEAS 2b cells potentially result in lack of induction of cytokines, such as proinflammatory cytokines and IFNs, following the expression of viral proteins. Future studies should therefore focus on defining the upstream cellular sensors and signalling intermediates responsible for IFN-λ induction by these viral proteins, to further delineate the early events that initiate this immune evasion cascade.

To investigate the importance of IFN-λ1/3 produced by MERS-CoV-nsp2 and SARS-CoV-1-nsp14 in IFN-α unresponsiveness, A549 cells were pretreated with IFN-λ1/3 to mimic the cellular environment after expression of MERS-CoV-nsp2 and SARS-CoV-1-nsp14. Further pretreatment of IFN-λ1/3 in A549 cells showed increased USP18 protein levels and attenuated responses in pSTAT1 and *MxA* induction after treatment with exogenous IFN-α. However, attenuated responses in pSTAT1 and ISG induction were not observed after treatment with exogenous IFN-β. One recent study identified a novel pathway of IFN-β, with IFN-β signalling through IFNAR1 in the absence of IFNAR2, while IFN-α requires IFNAR2 to engage IFNAR1 (46). Moreover, it has been reported that IFN-β binds IFNAR1 with a significantly higher affinity than IFN-α (47). As USP18 inhibits JAK/STAT signalling by disrupting the interaction between JAK1 and IFNAR2, this may explain why the JAK/STAT signalling pathway was still proficiently activated by IFN-β, even though USP18 levels were elevated following pretreatment with IFN-λ1/3. These combined features include higher receptor affinity and potentially unique receptor complex formation, likely allowing IFN-β to bypass USP18-mediated inhibition. In the last part of this study, we explored the mechanism behind the diminished response to IFN-α treatment following IFN-λ1/3 pretreatment in A549 cells. Subsequent to the stimulation of IFN-λ1/3, the upregulation of USP18 was evident. However, upon silencing USP18, enhanced pSTAT1 and *MxA* induction was observed, indicating an increased response to IFN-α. Consistent with previous studies, hepatocytes pretreated with IFN-λ1/3, IFN-λ2, or IFN-λ4 also exhibited attenuated IFN-α responses (29). Collectively, these findings indicate that IFN-λ1/3-induced USP18 selectively impairs IFN-α signalling while sparing IFN-β due to differences in receptor binding and signalling requirements, providing a mechanistic basis for IFN subtype-specific antiviral modulation.

Although our experimental data focused on MERS-CoV and SARS-CoV-1 proteins, the IFN-λ/USP18 axis is likely conserved in SARS-CoV-2. SARS-CoV-2 infection induces IFN-λ in epithelial cells, and viral proteins such as ORF6, nsp1, and nsp14 suppress type I IFN responses (48–50). Given USP18’s conserved role as a negative regulator of IFNAR signalling, SARS-CoV-2 may similarly exploit the IFN-λ/USP18 axis to attenuate IFN-α responsiveness while allowing IFN-β signalling to persist, providing a mechanism to evade early antiviral defenses without triggering excessive inflammation. These observations highlight USP18 as a conserved host factor co-opted by Coronaviruses (CoVs) and as a potential target for selective modulation to restore antiviral IFN responses.

Taken together, this study clearly demonstrated that MERS-CoV-nsp2 and SARS-CoV-1-nsp14 induced IFN-λ1/3, which potently blocked the IFN-α JAK/STAT signalling via upregulation of USP18 in A549 cells. Unlike A549 cells, expression of MERS-CoV-nsp2 and SARS-CoV-1-nsp14 in BEAS 2b cells induced USP18 expression, independent of the presence of IFN-λ1/3, to inhibit the IFN-α JAK/STAT signalling. These results explain why A549 and BEAS 2b cells expressing MERS-CoV-nsp2 and SARS-CoV-1-nsp14 respond poorly to IFN-α treatment. The further restoration of IFN-α responsiveness in both A549 and BEAS 2b cells following the silencing of USP18, suggests that USP18 modulates the antiviral IFN-α JAK/STAT signalling response. These findings shed light on the diminished efficacy of IFN-α-based treatment in patients infected with MERS-CoV and SARS-CoV-1, and highlights USP18 as a possible therapeutic target for the treatment of MERS-CoV and SARS-CoV-1 infection.

## Data Availability

The original contributions presented in the study are included in the article/supplementary material. Further inquiries can be directed to the corresponding author.

## References

[1] Sun HC Tang ZY Wang L Qin LX Ma ZC Ye QH . Postoperative interferon alpha treatment postponed recurrence and improved overall survival in patients after curative resection of HBV-related hepatocellular carcinoma: a randomized clinical trial. J Cancer Res Clin Oncol. (2006) 132:458–65. doi: 10.1007/s00432-006-0091-y, PMID: 16557381 PMC12161093

[2] Nguyen MH Wright TL . Therapeutic advances in the management of hepatitis B and hepatitis C. Curr Opin Infect Dis. (2001) 14:593–601. doi: 10.1097/00001432-200110000-00014, PMID: 11964881

[3] Rockley PF Tyring SK . Interferons alpha, beta and gamma therapy of anogenital human papillomavirus infections. Pharmacol Ther. (1995) 65:265–87. doi: 10.1016/0163-7258(94)00063-9, PMID: 7792318

[4] Krown SE Li P Von Roenn JH Paredes J Huang J Testa MA . Efficacy of low-dose interferon with antiretroviral therapy in Kaposi’s sarcoma: a randomized phase II AIDS clinical trials group study. J Interferon Cytokine Res. (2002) 22:295–303. doi: 10.1089/107999002753675712, PMID: 12034036

[5] Shalhoub S Farahat F Al-Jiffri A Simhairi R Shamma O Siddiqi N . IFN-α2a or IFN-β1a in combination with ribavirin to treat Middle East respiratory syndrome coronavirus pneumonia: a retrospective study. J Antimicrobial Chemotherapy. (2015) 70:2129–32. doi: 10.1093/jac/dkv085, PMID: 25900158 PMC7202429

[6] Cinatl J Morgenstern B Bauer G Chandra P Rabenau H Doerr HW . Treatment of SARS with human interferons. Lancet. (2003) 362:293–4. doi: 10.1016/S0140-6736(03)13973-6, PMID: 12892961 PMC7112413

[7] Zhao Z Zhang F Xu M Huang K Zhong W Cai W . Description and clinical treatment of an early outbreak of severe acute respiratory syndrome (SARS) in Guangzhou, PR China. J Med Microbiol. (2003) 52:715–20. doi: 10.1099/jmm.0.05320-0, PMID: 12867568

[8] Frieman M Yount B Heise M Kopecky-Bromberg SA Palese P Baric RS . Severe acute respiratory syndrome coronavirus ORF6 antagonizes STAT1 function by sequestering nuclear import factors on the rough endoplasmic reticulum/Golgi membrane. J Virol. (2007) 81:9812–24. doi: 10.1128/JVI.01012-07, PMID: 17596301 PMC2045396

[9] Wathelet MG Orr M Frieman MB Baric RS . Severe acute respiratory syndrome coronavirus evades antiviral signaling: role of nsp1 and rational design of an attenuated strain. J Virol. (2007) 81:11620–33. doi: 10.1128/JVI.00702-07, PMID: 17715225 PMC2168762

[10] Minakshi R Padhan K Rani M Khan N Ahmad F Jameel S . The SARS Coronavirus 3a protein causes endoplasmic reticulum stress and induces ligand-independent downregulation of the type 1 interferon receptor. PloS One. (2009) 4:e8342. doi: 10.1371/journal.pone.0008342, PMID: 20020050 PMC2791231

[11] Yang Y Zhang L Geng H Deng Y Huang B Guo Y . The structural and accessory proteins M, ORF 4a, ORF 4b, and ORF 5 of Middle East respiratory syndrome coronavirus (MERS-CoV) are potent interferon antagonists. Protein Cell. (2013) 4:951–61. doi: 10.1007/s13238-013-3096-8, PMID: 24318862 PMC4875403

[12] Wang W Xu L Su J Peppelenbosch MP Pan Q . Transcriptional regulation of antiviral interferon-stimulated genes. Trends Microbiol. (2017) 25:573–84. doi: 10.1016/j.tim.2017.01.001, PMID: 28139375 PMC7127685

[13] Zhang Y Gargan S Roche FM Frieman M Stevenson NJ . Inhibition of the IFN-α JAK/STAT pathway by MERS-CoV and SARS-CoV-1 proteins in human epithelial cells. Viruses. (2022) 14:667. doi: 10.3390/v14040667, PMID: 35458397 PMC9032603

[14] Marchetti M Monier M-N Fradagrada A Mitchell K Baychelier F Eid P . Stat-mediated signaling induced by type I and type II interferons (IFNs) is differentially controlled through lipid microdomain association and clathrin-dependent endocytosis of IFN receptors. Mol Biol Cell. (2006) 17:2896–909. doi: 10.1091/mbc.e06-01-0076, PMID: 16624862 PMC1483027

[15] Zanin N Viaris de Lesegno C Lamaze C Blouin CM . Interferon receptor trafficking and signaling: Journey to the cross roads. Front Immunol. (2021) 11:615603. doi: 10.3389/fimmu.2020.615603, PMID: 33552080 PMC7855707

[16] Kershaw NJ Murphy JM Liau NP Varghese LN Laktyushin A Whitlock EL . SOCS3 binds specific receptor–JAK complexes to control cytokine signaling by direct kinase inhibition. Nat Struct Mol Biol. (2013) 20:469–76. doi: 10.1038/nsmb.2519, PMID: 23454976 PMC3618588

[17] Liau NP Laktyushin A Lucet IS Murphy JM Yao S Whitlock E . The molecular basis of JAK/STAT inhibition by SOCS1. Nat Commun. (2018) 9:1558. doi: 10.1038/s41467-018-04013-1, PMID: 29674694 PMC5908791

[18] Schneider WM Chevillotte MD Rice CM . Interferon-stimulated genes: a complex web of host defenses. Annu Rev Immunol. (2014) 32:513–45. doi: 10.1146/annurev-immunol-032713-120231, PMID: 24555472 PMC4313732

[19] Böhmer F-D Friedrich K . Protein tyrosine phosphatases as wardens of STAT signaling. Jak-stat. (2014) 3:e28087. doi: 10.4161/jkst.28087, PMID: 24778927 PMC3995736

[20] Shuai K Liu B . Regulation of JAK–STAT signalling in the immune system. Nat Rev Immunol. (2003) 3:900–11. doi: 10.1038/nri1226, PMID: 14668806

[21] Horvath CM . Weapons of STAT destruction: Interferon evasion by paramyxovirus V proteins. Eur J Biochem. (2004) 271:4621–8. doi: 10.1111/j.1432-1033.2004.04425.x, PMID: 15606749

[22] Zheng J Yang P Tang Y Pan Z Zhao D . Respiratory syncytial virus nonstructural proteins upregulate SOCS1 and SOCS3 in the different manner from endogenous IFN signaling. J Immunol Res. (2015) 2015:738547. doi: 10.1155/2015/738547, PMID: 26557722 PMC4628668

[23] Ye H Duan X Yao M Kang L Li Y Li S . USP18 mediates interferon resistance of dengue virus infection. Front Microbiol. (2021) 12:682380. doi: 10.3389/fmicb.2021.682380, PMID: 34017322 PMC8130619

[24] Liu T Feng M Wen Z He Y Lin W Zhang M . Comparison of the characteristics of cytokine storm and immune response induced by SARS-CoV, MERS-CoV, and SARS-CoV-2 infections. J Inflammation Res. (2021) 14:5475. doi: 10.2147/JIR.S329697, PMID: 34720596 PMC8550203

[25] Au-Yeung N Mandhana R Horvath CM . Transcriptional regulation by STAT1 and STAT2 in the interferon JAK-STAT pathway. Jak-stat. (2013) 2:e23931. doi: 10.4161/jkst.23931, PMID: 24069549 PMC3772101

[26] Sung PS Cheon H Cho CH Hong S-H Park DY Seo H-I . Roles of unphosphorylated ISGF3 in HCV infection and interferon responsiveness. Proc Natl Acad Sci. (2015) 112:10443–8. doi: 10.1073/pnas.1513341112, PMID: 26216956 PMC4547285

[27] Fujiwara T Oda K Yokota S Takatsuki A Ikehara Y . Brefeldin A causes disassembly of the Golgi complex and accumulation of secretory proteins in the endoplasmic reticulum. J Biol Chem. (1988) 263:18545–52. doi: 10.1016/S0021-9258(19)81393-5, PMID: 3192548

[28] François-Newton V Magno de Freitas Almeida G Payelle-Brogard B Monneron D Pichard-Garcia L Piehler J . USP18-based negative feedback control is induced by type I and type III interferons and specifically inactivates interferon α response. PloS One. (2011) 6:e22200. doi: 10.1371/journal.pone.0022200, PMID: 21779393 PMC3136508

[29] Sung PS Hong S-H Chung J-H Kim S Park S-H Kim HM . IFN-λ4 potently blocks IFN-α signalling by ISG15 and USP18 in hepatitis C virus infection. Sci Rep. (2017) 7:3821. doi: 10.1038/s41598-017-04186-7, PMID: 28630501 PMC5476576

[30] Andreakos E Salagianni M Galani IE Koltsida O . Interferon-λs: Front-line guardians of immunity and homeostasis in the respiratory tract. Front Immunol. (2017) 8:1232. doi: 10.3389/fimmu.2017.01232, PMID: 29033947 PMC5626824

[31] Honke N Shaabani N Zhang D-E Hardt C Lang KS . Multiple functions of USP18. Cell Death Dis. (2016) 7:e2444–4. doi: 10.1038/cddis.2016.326, PMID: 27809302 PMC5260889

[32] Munnur D Banducci-Karp A Sanyal S . ISG15 driven cellular responses to virus infection. Biochem Soc Trans. (2022) 50:1837–46. doi: 10.1042/BST20220839, PMID: 36416643 PMC9788361

[33] Xiao C Qin B Chen L Liu H Zhu Y Lu X . Preactivation of the interferon signalling in liver is correlated with nonresponse to interferon alpha therapy in patients chronically infected with hepatitis B virus. J Viral Hepatitis. (2012) 19:e1–e10. doi: 10.1111/j.1365-2893.2011.01471.x, PMID: 22239505

[34] Li L Lei Q-S Zhang S-J Kong L-N Qin B . Suppression of USP18 potentiates the anti-HBV activity of interferon alpha in HepG2. 2.15 cells via JAK/STAT signaling. PloS One. (2016) 11:e0156496. doi: 10.1371/journal.pone.0156496, PMID: 27227879 PMC4882066

[35] Randall G Chen L Panis M Fischer AK Lindenbach BD Sun J . Silencing of USP18 potentiates the antiviral activity of interferon against hepatitis C virus infection. Gastroenterology. (2006) 131:1584–91. doi: 10.1053/j.gastro.2006.08.043, PMID: 17101330

[36] Qian G Zhu L Li G Liu Y Zhang Z Pan J . An integrated view of deubiquitinating enzymes involved in type I interferon signaling, host defense and antiviral activities. Front Immunol. (2021) 12:742542. doi: 10.3389/fimmu.2021.742542, PMID: 34707613 PMC8542838

[37] Goldmann T Zeller N Raasch J Kierdorf K Frenzel K Ketscher L . USP 18 lack in microglia causes destructive interferonopathy of the mouse brain. EMBO J. (2015) 34:1612–29. doi: 10.15252/embj.201490791, PMID: 25896511 PMC4475397

[38] Kotenko SV Rivera A Parker D Durbin JE . Type III IFNs: beyond antiviral protection. Semin Immunol. (2019) 43:101303. doi: 10.1016/j.smim.2019.101303, PMID: 31771761 PMC7141597

[39] Kotenko SV Gallagher G Baurin VV Lewis-Antes A Shen M Shah NK . IFN-λs mediate antiviral protection through a distinct class II cytokine receptor complex. Nat Immunol. (2003) 4:69–77. doi: 10.1038/ni875, PMID: 12483210

[40] Lazear HM Nice TJ Diamond MS . Interferon-λ: immune functions at barrier surfaces and beyond. Immunity. (2015) 43:15–28. doi: 10.1016/j.immuni.2015.07.001, PMID: 26200010 PMC4527169

[41] Wells AI Coyne CB . Type III interferons in antiviral defenses at barrier surfaces. Trends Immunol. (2018) 39:848–58. doi: 10.1016/j.it.2018.08.008, PMID: 30219309 PMC6179363

[42] Bisanz JE Suppiah P Thomson WM Milne T Yeoh N Nolan A . The oral microbiome of patients with axial spondyloarthritis compared to healthy individuals. PeerJ. (2016) 4:e2095. doi: 10.7717/peerj.2095, PMID: 27330858 PMC4906644

[43] Qian Z Travanty EA Oko L Edeen K Berglund A Wang J . Innate immune response of human alveolar type ii cells infected with severe acute respiratory syndrome–coronavirus. Am J Respir Cell Mol Biol. (2013) 48:742–8. doi: 10.1165/rcmb.2012-0339OC, PMID: 23418343 PMC3727876

[44] Sheets PL Yost GS Carlson GP . Benzene metabolism in human lung cell lines BEAS-2B and A549 and cells overexpressing CYP2F1. J Biochem Mol Toxicol. (2004) 18:92–9. doi: 10.1002/jbt.20010, PMID: 15122651

[45] Hillyer P Shepard R Uehling M Krenz M Sheikh F Thayer KR . Differential responses by human respiratory epithelial cell lines to respiratory syncytial virus reflect distinct patterns of infection control. J Virol. (2018) 92:e02202‑17. doi: 10.1128/jvi.02202-02217, PMID: 29769339 PMC6052282

[46] Kaur S Platanias LC . IFN-β-specific signaling via a unique IFNAR1 interaction. Nat Immunol. (2013) 14:884–5. doi: 10.1038/ni.2686, PMID: 23959176

[47] Schreiber G . The molecular basis for differential type I interferon signaling. J Biol Chem. (2017) 292:7285–94. doi: 10.1074/jbc.R116.774562, PMID: 28289098 PMC5418031

[48] Lei X Dong X Ma R Wang W Xiao X Tian Z . Activation and evasion of type I interferon responses by SARS-CoV-2. Nat Commun. (2020) 11:3810. doi: 10.1038/s41467-020-17665-9, PMID: 32733001 PMC7392898

[49] Xia H Cao Z Xie X Zhang X Chen JY-C Wang H . Evasion of type I interferon by SARS-CoV-2. Cell Rep. (2020) 33:108234. doi: 10.1016/j.celrep.2020.108234, PMID: 32979938 PMC7501843

[50] Galani I-E Rovina N Lampropoulou V Triantafyllia V Manioudaki M Pavlos E . Untuned antiviral immunity in COVID-19 revealed by temporal type I/III interferon patterns and flu comparison. Nat Immunol. (2021) 22:32–40. doi: 10.1038/s41590-020-00840-x, PMID: 33277638

